# Intraspecific Variation of Transposable Elements Reveals Differences in the Evolutionary History of Fungal Phytopathogen Pathotypes

**DOI:** 10.1093/gbe/evad206

**Published:** 2023-11-17

**Authors:** Anne A Nakamoto, Pierre M Joubert, Ksenia V Krasileva

**Affiliations:** Department of Plant and Microbial Biology, University of California, Berkeley, California, USA; Department of Plant and Microbial Biology, University of California, Berkeley, California, USA; Department of Plant and Microbial Biology, University of California, Berkeley, California, USA

**Keywords:** comparative genomics, fungal plant pathogens, *Magnaporthe oryzae*, rice blast, transposable elements, intraspecific variation

## Abstract

Transposable elements (TEs) contribute to intraspecific variation and play important roles in the evolution of fungal genomes. However, our understanding of the processes that shape TE landscapes is limited, as is our understanding of the relationship between TE content, population structure, and evolutionary history of fungal species. Fungal plant pathogens, which often have host-specific populations, are useful systems in which to study intraspecific TE content diversity. Here, we describe TE dynamics in five lineages of *Magnaporthe oryzae*, the fungus that causes blast disease of rice, wheat, and many other grasses. We identified differences in TE content across these lineages and showed that recent lineage-specific expansions of certain TEs have contributed to overall greater TE content in rice-infecting and *Setaria*-infecting lineages. We reconstructed the evolutionary histories of long terminal repeat-retrotransposon expansions and found that in some cases they were caused by complex proliferation dynamics of one element and in others by multiple elements from an older population of TEs multiplying in parallel. Additionally, we found evidence suggesting the recent transfer of a DNA transposon between rice- and wheat-infecting *M. oryzae* lineages and a region showing evidence of homologous recombination between those lineages, which could have facilitated such a transfer. By investigating intraspecific TE content variation, we uncovered key differences in the proliferation dynamics of TEs in various pathotypes of a fungal plant pathogen, giving us a better understanding of the evolutionary history of the pathogen itself.

**Significance**

Transposable elements (TEs) are known to play a major role in fungal genome evolution and intraspecific variation, yet the processes that shape TE content diversity in the context of species evolutionary history are not well understood. By characterizing complex TE expansion dynamics in *Magnaporthe oryzae*, we found that different lineages of this important fungal plant pathogen experienced distinct evolutionary histories. Our findings demonstrate that studying TE dynamics can lead to a better understanding of intraspecific variation, which is especially important in distinguishing closely related host-specific fungal pathogen populations.

## Introduction

Many fungal species display extensive intraspecific variation, allowing them to adapt to a wide range of lifestyles and environments (Mathieu et al. 2018; Branco 2019; Monte et al. 2021). Transposable elements (TEs), a diverse collection of repetitive, mobile sequences, are known to generate genomic diversity and contribute to genome evolution (Wells and Feschotte 2020; Almojil et al. 2021). Along with the substantial diversity of TE content across fungal species (Raffaele and Kamoun 2012; Castanera et al. 2016; Muszewska et al. 2019), there are examples of intraspecific TE content variation in edible mushrooms, fungal pathogens, mycorrhizal fungi, and yeast (Castanera et al. 2016; Shirke et al. 2016; Chen et al. 2018; Bleykasten-Grosshans et al. 2021; Oggenfuss et al. 2021; Gourlie et al. 2022). Yet, we are only beginning to understand how differences in TE content arise in such systems and how this may reflect a species’ evolutionary history. Fungal plant pathogens provide interesting models for investigating TE content diversity, as many have host-specific populations (Möller and Stukenbrock 2017). Many of these fungi are also thought to have a “two-speed” genome structure, where slowly evolving, gene-rich regions are separated from rapidly evolving regions with many TEs and few genes (Dong et al. 2015; Faino et al. 2016). Disease-causing effector genes are thought to undergo rapid gain, loss, and evolution in genomic regions associated with TEs, which can benefit the pathogen by allowing evasion of their host's immune response (Sánchez-Vallet et al. 2018). Although some studies have identified intraspecific differences in fungal plant pathogen TE content (Shirke et al. 2016; Oggenfuss et al. 2021; Gourlie et al. 2022), the relationship between TEs and the evolutionary histories of these species remains largely uncharacterized.

**Fig. 1.**
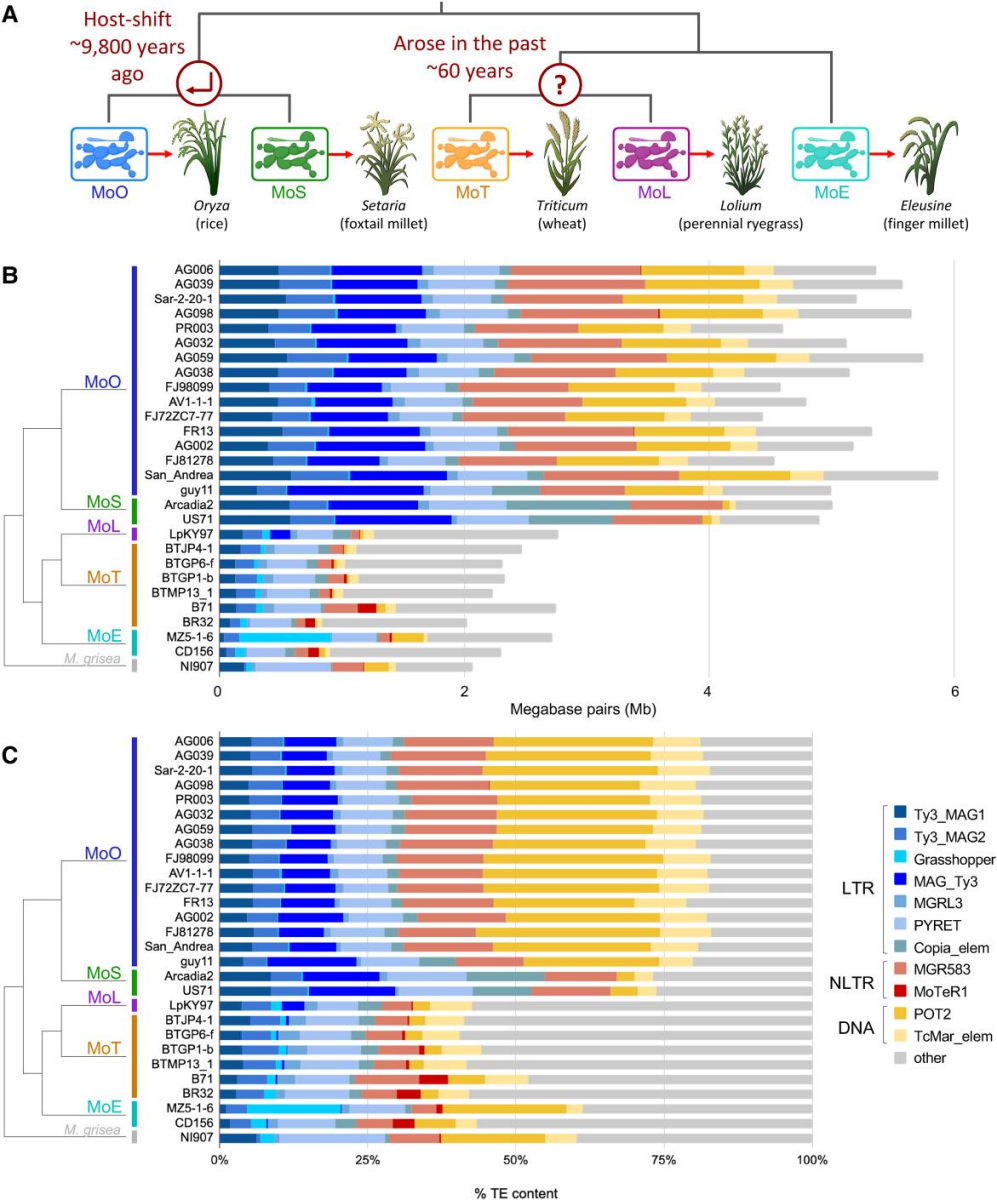
Large variation of TE content exists between *M. oryzae* genomes of different lineages. (*A*) Current understanding of relationships between different host-infecting *M. oryzae* lineages. Icons representing *M. oryzae* are from BioRender.com. (*B*) Stacked bar plot showing the number of megabase pairs (Mb) each TE occupies in each genome. (*C*) Stacked bar plot showing the percentage that each TE family makes up out of all TEs in each genome. At the left of both plots is the lineage each genome belongs to and the evolutionary relationships between lineages (Gladieux, Condon, et al. 2018) (branch lengths not to scale). Names of the TE families and their classification are shown in the key. LTR, long terminal repeat-retrotransposon; NLTR, non–LTR-retrotransposon; DNA, DNA transposon.

*Magnaporthe oryzae* is an important fungal pathogen that causes the blast disease of various grasses, including important crops such as rice and wheat (Dean et al. 2012: 10). *Magnaporthe oryzae's* wide host range is associated with substantial intraspecific diversity. The species is composed of distinct pathotypes that include lineages infecting *Oryza* (rice), *Setaria* (foxtail), *Triticum* (wheat), *Lolium* (ryegrass), and *Eleusine* (goosegrass) (MoO, MoS, MoT, MoL, and MoE, respectively) (Gladieux, Condon, et al. 2018). All lineages are thought to have arisen recently, yet there is large variation in their relative ages. It is well accepted that the closely related MoO and MoS lineages diverged from their common ancestor around the time of rice domestication, ∼9,800 years ago via a host shift of MoS to rice (Couch et al. 2005; Gladieux, Ravel, et al. 2018; Zhong et al. 2018). However, wheat blast was discovered much more recently in 1985 (Singh et al. 2021) and was thought to have arisen via a host shift of MoL to wheat (Inoue et al. 2017; Ceresini et al. 2019). Alternatively, a recent study suggests that a large admixture event involving recombination between isolates of multiple pathotypes, including MoE and a relative of MoO and MoS, may have given rise to the closely related MoT and MoL within the past 60 years (Rahnama et al. 2021). It is clear that we have yet to fully understand the processes shaping the present structure of the *M. oryzae* lineages (fig. 1*A*).

Previous studies have shown that TEs can have variable content in and major effects on the *M. oryzae* genome and its host specificity. Major TE families previously found in *M. oryzae* include *POT2*, *PYRET*, *MAGGY*, *MGRL*, *OCCAN*, *MgSINE*, *GYMAG1*, and *GYMAG2*, where *MAGGY*, *PYRE*T, and *POT2* have shown copy number variation in different *M. oryzae* isolates (Shirke et al. 2016). Insertion of these TEs has been shown to affect pathogenicity, for example, the *POT2* DNA transposon insertion into the *AVR-Pib* effector gene of MoO isolates allowed them to evade recognition by the *Pib* gene in rice, overcoming resistance (Li et al. 202 3). Additionally, it is hypothesized that TE insertions caused the functional loss of the *PWT3* effector, enabling a host jump of MoL to wheat (Inoue et al. 2017). Many studies have also shown that *M. oryzae* experiences frequent gene gains and losses, often in association with TEs (Shirke et al. 2016; Yoshida et al. 2016; Thierry et al. 2022; Joubert and Krasileva 2023). Finally, extrachromosomal circular DNAs have been shown to confer great adaptive potential (Paulsen et al. 2018), and *M. oryzae* was found to produce a large set of eccDNAs consisting of many long terminal repeat (LTR)-retrotransposon sequences (Joubert and Krasileva 2022). Although TEs are associated with many adaptive processes in *M. oryzae*, the relationship between TE content variation and the population structure and evolutionary history of the species remains unknown.

In this study, we compared TE content and proliferation dynamics across the *M. oryzae* lineages. We assembled an unbiased library of TEs and produced robust annotations, which revealed striking differences in overall TE content between lineages. We observed that recent lineage-specific expansions of LTR-retrotransposons have contributed to greater TE content in MoO and MoS. The histories and dynamics of these expansions were complex. Some were caused by the proliferation of one element, whereas others consisted of multiple elements from an older population of TEs that proliferated in parallel. Additionally, we found evidence suggesting a recent transfer of a DNA transposon between MoO and MoT and found a potential region of recombination between those lineages that could have facilitated such a transfer. Together, these results showed complex TE expansion dynamics in *M. oryzae* lineages that shaped *M. oryzae's* evolutionary history.

## Results

### TE Content in *M. oryzae* Varies Greatly Across Lineages and Isolates

To analyze TE content in *M. oryzae*, we first constructed a library representative of TE diversity in all lineages. Since highly contiguous genome assemblies provide the most complete and accurate view of TE content (Rech et al. 2022), a set of 27 *M. oryzae* genomes of various lineages, including available chromosome level assemblies and others with <75 contigs, was gathered from National Center for Biotechnology Information (NCBI) GenBank (supplementary table S1, Supplementary Material online). We then constructed a pipeline based on previous methods (Muszewska et al. 2019) to annotate TEs. The pipeline (supplementary fig. S1, Supplementary Material online) utilized one representative genome from each lineage to perform de novo repeat annotation. We then added the RepBase (Bao et al. 2015) library of known TEs in fungi and filtered the combined library against a list of TE-associated protein domains (Muszewska et al. 2019). This ensured that only potentially active elements, which could have contributed to the recent evolutionary history of lineage formation, were kept, thus excluding highly degenerated and nonautonomous elements (supplementary table S2, Supplementary Material online). The library was further refined by manual classification of elements using protein domain-based phylogenies (supplementary fig. S1, Supplementary Material online). Elements that formed a subclade with a known RepBase TE were classified as being part of the same family. This resulted in the classification of many de novo elements as members of known families (*Ty3_MAG1*, *Ty3_MAG2*, *Grasshopper*, *MAG_Ty3*, *MGRL3*, *PYRET*, *MGR583*, *MoTeR1*, and *POT2*) or as new families part of a known TE superfamily (*Copia_elem* and *TcMar_elem*) (supplementary table S3, Supplementary Material online). TEs that did not group in a subclade containing a known family or superfamily were classified as “unknown” and were largely made up of singleton or low copy number families (Additional File 2). We then used the classified library to annotate TEs in our set of *M. oryzae* genomes (supplementary table S1, Supplementary Material online) and verified that each hit contained a TE-associated domain. This approach provided high-quality and unbiased copy number and positional information of TEs in each genome.

Using our TE annotations, we observed striking differences in TE content between genomes of different lineages, and these differences seemed to follow the evolutionary relationships between lineages (fig. 1, supplementary fig. S2, Supplementary Material online). Most notably, MoO and MoS genomes contained much higher TE content than MoT, MoL, and MoE (fig. 1*B*). In MoO and MoS, an average of 11.14% (5.1 Mb) of the genome consisted of annotated TEs, whereas the average was 5.44% (2.4 Mb) for the other three lineages (supplementary Table S1, Supplementary Material online, fig. 1*B*). We observed that principal component analysis (PCA) of TE content also clearly separates the lineages (supplementary fig. S3*A*–*C*, Supplementary Material online). The *Magnaporthe grisea* isolate that was used as an outgroup had very similar TE content to the MoT, MoL, and MoE isolates, suggesting that the MoO-MoS clade may have acquired its higher TE content after diverging from the other lineages (fig. 1*B*). An analysis of the genome sizes of the isolates showed that the increased TE content in MoO-MoS was not due to genome duplication (supplementary table S1 and fig. S3*D*, Supplementary Material online). Although we did observe a correlation between TE content and genome size, most of the signal appears to come from MoO and MoS isolates (supplementary fig. S3*D*, Supplementary Material online). Furthermore, correlation tests showed that the differences we observed between lineages were not due to assembly quality or completeness, whereas TE content was highly correlated with the lineage an isolate belonged to (supplementary fig. S3*E*, Supplementary Material online). Finally, we observed differences in the relative proportions of certain annotated families. For example, *MAG_Ty3* content was proportionally greater than other lineages in MoO and *Copia_elem* content was proportionally greater than other lineages in MoS (fig. 1*C*). These differences in TE family prevalence across lineages hinted at complex lineage-specific TE dynamics, rather than genome-wide contraction or expansion of all TE content.

Although there was an overall greater number of TEs in MoO and MoS, some families were more prevalent in the other lineages or in individual genomes. The *Grasshopper* LTR-retrotransposon made up a large portion of TE content in the MoE MZ5-1-6 genome specifically, but much less in the other MoE, MoT, and MoL genomes, and was absent in MoO and MoS. Additionally, the *MoTeR1* family had greater copy number in MoT's B71 and BR32, and MoE's CD156, but less in the other MoT, MoL, and MoE genomes, and was also absent from MoO and MoS (fig. 1*C*). This indicated that although MoT, MoL, and MoE have lower TE content, they may be more prone to isolate-specific TE dynamics, in contrast to the larger and more uniform TE content in MoO and MoS.

### Recent Lineage-Specific Expansions of LTR-Retrotransposons Led to Differences in TE Content Between *M. oryzae* Lineages

**Fig. 2.**
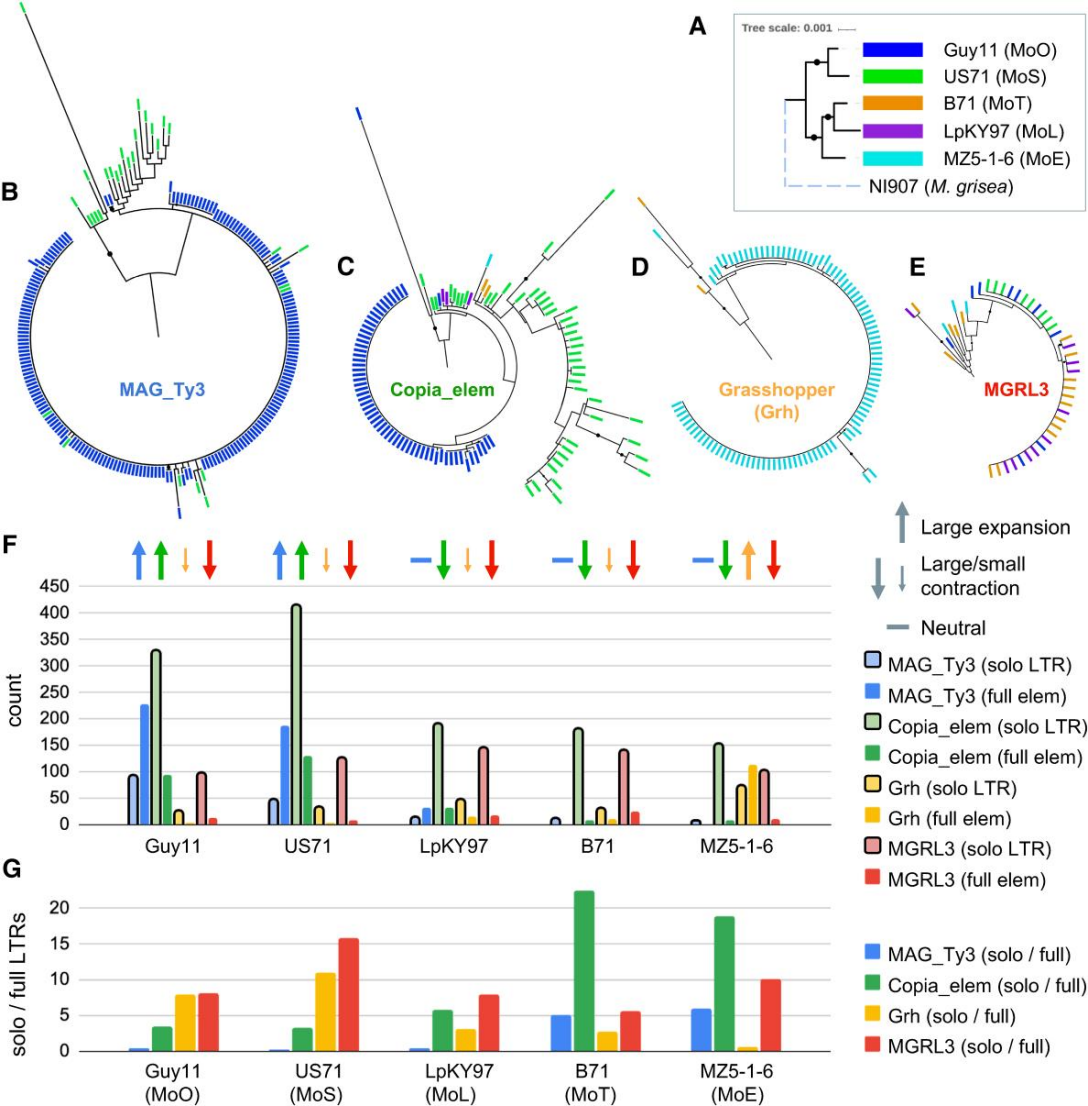
Certain LTR-retrotransposons have experienced lineage-specific expansions. (*A*) ML phylogeny of representative genomes of each lineage, based on the alignment of 8,655 SCOs. Branch lengths are to scale, except for the dashed line of the *M. grisea* outgroup. Bootstrap value of 100 is indicated by black circles. Domain-based ML phylogenies of TEs (*B*) *MAG_Ty3*, (*C*) *Copia_elem*, (*D*) *Grasshopper*, and (*E*) *MGRL3* are shown. Colored rectangle tips correspond to the genome each element is from, as shown in (A), and black circles indicate bootstrap value ≥80. (*F*) In this barchart, solo-LTR copy number is compared with the number of full-length TEs to represent the expansion and contraction dynamics of TE families in each genome. Each bar is colored according to the specific family, corresponding to the color of the label within each TE phylogeny (*B*–*E*). Lighter bars outlined in black represent solo-LTRs. Arrows above each set of bars indicate our interpretation of the predominant explanation (expansion vs. contraction) for the lineage-specific differences observed, based on the number of full elements versus solo-LTRs within the genome, and comparison of the number of full elements and solo-LTRs across all genomes. (*G*) Barchart showing the ratio of solo-LTR copy number to full-length element copy number for each LTR-retrotransposon family in each genome, with the same color-coding as in previous parts.

We next tested whether genome-wide or TE specific contraction or expansion dynamics led to the differences in TE content across lineages. The bulk of the differences between MoO-MoS and other lineages seemed to be explained by multiple LTR-retrotransposon families and the POT2 DNA transposon family (fig. 1*C*). We first focused on the LTR-retrotransposons and constructed domain-based maximum likelihood (ML) phylogenies (fig. 2*B*–*E*, supplementary fig. S4, Supplementary Material online) for each of the seven families using all copies annotated in the highest quality genome of each lineage. The TE trees were compared with the genome tree (fig. 2*A*), which was generated based on the alignment of 8,655 single-copy orthologous genes (SCOs), in order to compare evolutionary relationships. Based on our analysis, three LTR-retrotransposon families stood out as having experienced lineage-specific expansions. *MAG_Ty3* showed a large expansion in MoO and a smaller expansion in MoS (fig. 2*B*). *Copia_elem* expanded in both MoO and MoS (fig. 2*C*), and *Grasshopper* expanded only in the MoE MZ5-1-6 genome (fig. 2*D*). A helpful point of comparison was the *MGRL3* LTR-retrotransposon, which was present at a low copy number in all of the lineages. Elements in the *MGRL3* phylogeny did not strictly group by lineage and were more interleaved (fig. 2*E*), suggesting that it experienced an older expansion before the *M. oryzae* lineages diverged and has not proliferated recently. The other LTR-retrotransposon phylogenies of *Ty3_MAG1*, *Ty3_MAG2*, and *PYRET* (supplementary fig. S4, Supplementary Material online) also indicated older activity. These different histories show that a genome-wide deregulation of TEs was likely not responsible for the higher TE content in MoO and MoS but rather that it resulted from TE family-specific dynamics.

We then wanted to see whether *Grasshopper*, *MAG_Ty3*, and *Copia_elem* had experienced lineage-specific expansions that occurred after all *M. oryzae* lineages had diverged or whether expansions occurred in all lineages and were followed by subsequent losses. Since LTR-retrotransposons consist of an internal region that is flanked by direct repeats known as LTRs, they can be excised from the genome by nonallelic homologous recombination between the flanking LTRs (Wells and Feschotte 2020). When this occurs, a single LTR sequence known as a “solo-LTR” is left behind in the genome. We utilized the presence of these sequences to investigate TE expansion versus contraction dynamics. In general, a large number of solo-LTRs and few full elements would suggest contraction of an LTR-retrotransposon population, whereas few solo-LTRs and many full elements suggest expansion (Jedlicka et al. 2020). Although this ratio can be informative, it is also important to compare the raw copy numbers of solo and full LTRs across lineages to observe the baseline for what all lineages have in common. Then, expansion or contraction events unique to particular lineages can be inferred. By comparing the copy number of solo-LTRs and full elements across lineages, we observed that lineage-specific expansions are largely responsible for LTR-retrotransposon copy number variation, rather than the removal of these elements from some lineages (fig. 2*F*). *MAG_Ty3* had <13 solo-LTRs present in each of the MoL, MoT, and MoE lineages, which was much fewer than the 227 and 188 full-length *MAG_Ty3* in MoO and MoS, respectively. Thus, *MAG_Ty3's* higher copy number in MoO and MoS was likely due to expansion in those lineages only. The *Copia_elem* family had a lot more solo-LTRs present in all genomes (>150), suggesting that older expansions may have occurred before the divergence of the lineages and that they were then partially removed. However, there were many more *Copia_elem* solo-LTRs in MoO and MoS (>300) along with more full copies, which could only have been achieved by expansions unique to MoO and MoS. *Grasshopper* had <50 solo-LTRs in all genomes besides MoE MZ5-1-6, which had 114 full-length elements, indicating an expansion in that genome only. In contrast, *MGRL3* had a relatively high number of solo-LTRs (>96) and a low number of full elements (<26) in all lineages. Additionally, the ratio of solo-LTRs to full element copy number was consistently high for MGRL3, whereas it varied across genomes more greatly for the other LTR-retrotransposon families (fig. 2*G*). This supports the idea that *MGRL3* was expanded before the divergence of the lineages and then was largely removed from all of them over time. Thus, although both expansion and contraction play roles in determining LTR-retrotransposon copy number in *M. oryzae*, large expansions were the predominant cause of lineage-specific and isolate-specific copy number variation for the LTR-retrotransposons of interest.

Since several effector structural groups are also expanded in *M. oryzae*, such as ART and MAX effectors (Seong and Krasileva 2021), we tested whether their expansion might be associated with the expansion of our TEs of interest. This would tell us whether specific TE expansions could have played a role in the expansion of *M. oryzae's* effector repertoire and thus the disease-causing abilities of different lineages. We performed permutation tests following previous methods (Joubert and Krasileva 2022) on the distance of ART and MAX effectors to TEs but did not find them to be closer to any particular TEs than other effectors (supplementary fig. S5, Supplementary Material online). Rather, it seems that all effectors, including ARTs and MAXs, are in general closer to all the expanded TEs of interest than other genes are (supplementary fig. S6, Supplementary Material online), indicating a more global pattern of two-speed genome compartmentalization (Dong et al. 2015; Faino et al. 2016).

### Complex LTR-Retrotransposon Proliferation History and Dynamics Explain Lineage-Specific Expansions in *M. oryzae*

**Fig. 3.**
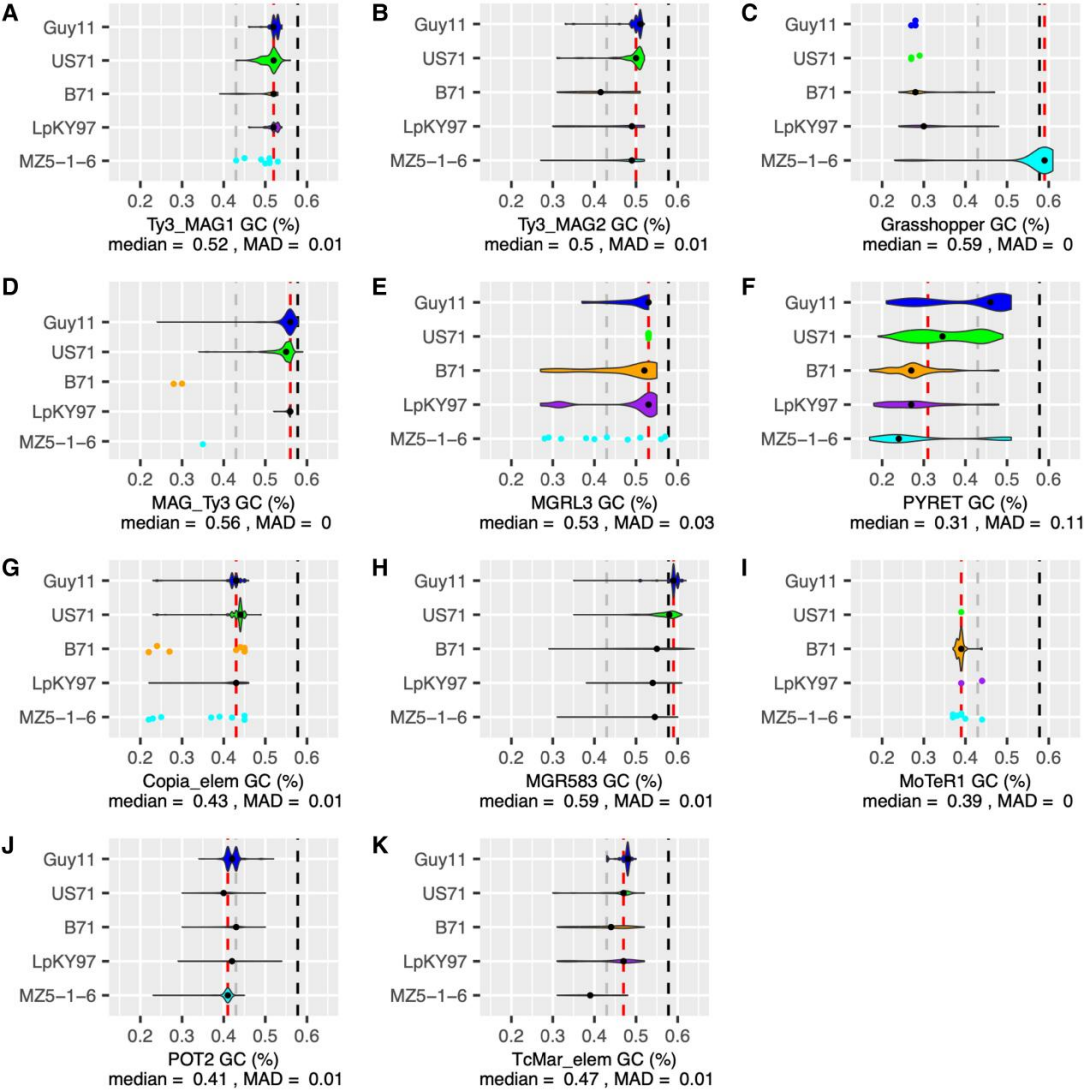
RIP has had little effect on recently expanded TE sequences. The GC content of each TE family in each representative genome is shown for (*A*) *Ty3_MAG1*, (*B*) *Ty3_MAG2*, (*C*) *Grasshopper*, (*D*) *MAG_Ty3*, (*E*) *MGRL3*, (*F*) *PYRET*, (*G*) *Copia_elem*, (*H*) *MGR583*, (*I*) *MoTeR1*, (*J*) *POT2*, and (*K*) *TcMar_elem*. Data are shown as a violin plot unless there are <10 points, in which case a jitter plot was used. Within each plot, violin width is proportional to the number of TE copies represented. Dashed lines indicate: black, genome-wide average GC content of coding sequences; gray, noncoding sequences; and red, the median GC content of the TE. The median GC content of the TE and the MAD is specified.

Next, we sought to better understand the timing and history of the LTR-retrotransposon expansions we observed. To do so, we used nucleotide sequence comparison and sequence divergence tests, which make the assumption that sequence divergence occurs at the same rate in all TEs. Thus, this assumption could be violated by the presence of repeat induced point mutation (RIP), a mutagenic mechanism in fungi that targets repetitive elements like TEs, causing GC to TA mutations (Pereira et al. 2021). RIP is only active during sexual reproduction (Ikeda et al. 2002), and previous studies have reported that it is minimally active in *M. oryzae* given its largely clonal life cycle (Ikeda et al. 2002; Pereira et al. 2021; van Wyk et al. 2021). However, there is evidence that RIP occurred during the large admixture event that potentially formed MoT and MoL (Rahnama et al. 2021), and RIP-associated genes *RID* and *DIM2* have been identified as present in some *M. oryzae* isolates (Bewick et al. 2019; van Wyk et al. 2021). An initial search of our orthogroups revealed that *RID* and *DIM2* were SCOs in our genomes, indicating the possibility of RIP affecting our TE divergence analyses. To measure how prevalent RIP was in our sequences, we calculated the GC content of all TE copies in each representative genome and compared them with the genome-wide average GC content of coding and noncoding regions (Pereira et al. 2021). We found that, although each TE family had a different median GC content, the individual copies did not deviate much from that value in most families (fig. 3). Although we observed some trailing copies with low GC content, these were likely older elements that had been affected by RIP in the past. The *Pyret* LTR-retrotransposon had a GC content distribution with a strong skew that suggested it may have experienced RIP (fig. 3*F*). However, our data indicated that *Pyret* had not been recently active, because it had a similar number of copies in each lineage (fig. 1*B*) and elements in its phylogeny did not group by lineage (supplementary fig. S4, Supplementary Material online), similar to *MGRL3*. Thus, RIP affecting Pyret had likely not occurred recently, and this family served as a good contrast to the recent LTR-retrotransposon expansions we focused on. Since the composite RIP index (CRI) is often used to assess the prevalence of RIP, we additionally used The RIPper tool (Van Wyk et al. 2019) to determine CRI per element in each genome (supplementary fig. S7, Supplementary Material online). Most of our recently expanded TEs of interest had median CRI of zero or less, indicating no RIP. Exceptions to this were *Copia_elem* in US71 and *POT2* in MZ5-1-6. However, higher CRI doesn’t necessarily mean that RIP occurred recently, as a previously RIPped element may have contributed to a recent expansion, and all copies would have similar CRI. As a final test, we compared the GC content of TEs in an MoO isolate originating from a recombining lineage (Guy11) with a clonal MoO isolate (FJ98099) (Latorre et al. 2020). There were no differences in the GC content between these two isolates, indicating very little RIP activity even in the sexually recombining MoO isolate (supplementary fig. S8, Supplementary Material online). These analyses strongly suggest that although RIP may have been active in the past, it has had little effect on recently expanded TEs, and so the divergence tests we performed on our recently expanded TEs were valid.

**Fig. 4.**
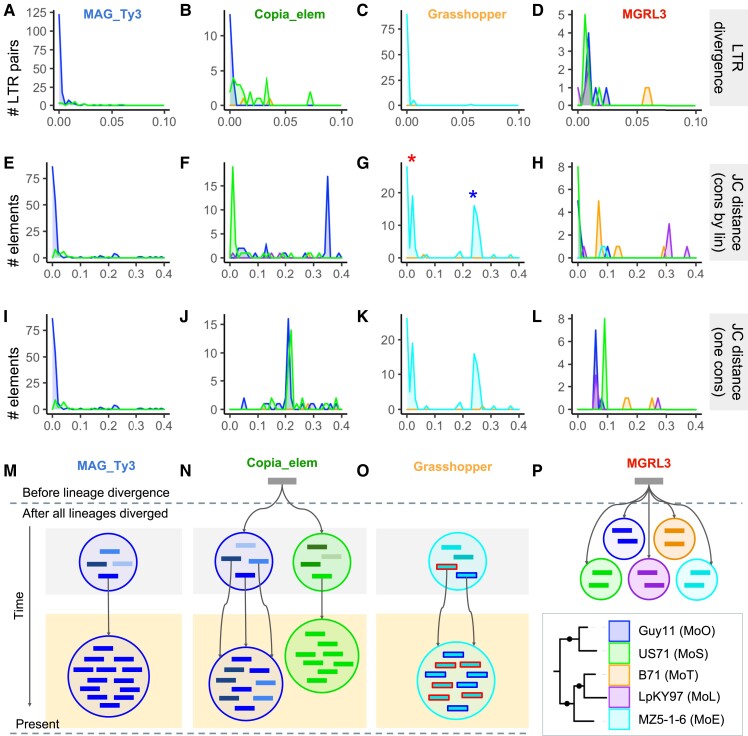
TE expansions in *M. oryzae* experienced complex histories that differ between various lineages. Columns correspond to *MAG_Ty3*, *Copia_elem*, *Grasshopper*, and *MGRL3* (from left to right, for each row of the figure). (*A*–*D*) Divergence between flanking LTR sequences of LTR-retrotransposons. (*E*–*H*) The Jukes–Cantor distance calculated using a separate consensus for each lineage. (*I*–*L***)** The Jukes–Cantor distance calculated using one consensus for all lineages. (*M*–*P***)** Schematic diagrams representing our hypothesis for the history of TE expansion events for each of the families. The representation of the current population for each TE is highlighted in yellow, and these expansions occurred either by one or multiple copies from an older population of TEs with sequence differences (highlighted in gray) proliferating recently. We indicate that TEs from different lineages might have proliferated from the same original copy for *Copia_elem* and *MGRL3*. Blue and red outlined *Grasshopper* rectangles correspond to the two labeled peaks in G. *MGRL3* is an example of an old expansion. The tree in the bottom right corner serves as a key for color-coding of the lineages for all parts of the figure.

To investigate the timing of LTR-retrotransposon expansions, we first sought to date individual TE insertions. A method for determining the age of LTR-retrotransposon insertions is to calculate the divergence between the flanking LTR sequences within an element (Jedlicka et al. 2020), because they are identical upon insertion (Wells and Feschotte 2020). Flanking LTRs of older elements would be more divergent, because they have had more time to accumulate mutations, whereas newer elements would have highly similar LTRs (Jedlicka et al. 2020). We determined LTR sequence divergence for *MAG_Ty3*, *Copia*, *Grasshopper*, and *MGRL3* retrotransposons (fig. 4*A*–*D*). Our results indicated that the expanded LTRs (*MAG_Ty3*, *Copia*, and *Grasshopper*) were inserted very recently, as many LTR pairs had zero sequence differences between them. The fact that LTR sequences are quite short (250–500 bp) combined with a reported mutation rate of 1.98e^−8^ substitutions/site/year in *M. oryzae* (Gladieux, Ravel, et al. 2018) likely contributed to this result. Nevertheless, given this mutation rate, a 500 bp sequence would be expected to have mutated once in the past 50,000 years, indicating that these expansions could have occurred at any point since then, including more recently than the divergence of the MoO and MoS lineages 9,800 years ago (Gladieux, Ravel, et al. 2018). The *Copia_elem* in the MoS genome, on the other hand, showed a broader range of LTR divergence values, indicating proliferation events spread out over time (fig. 4*B*). *MGRL3* had slightly higher divergences between its flanking LTRs that were generally similar for all the lineages (fig. 4*D*), supporting the idea that it experienced an older expansion in a single period before the divergence of the lineages. Overall, these findings support our interpretation that the lineage-specific LTR-retrotransposon expansions occurred recently.

Next, using the Jukes–Cantor distance metric, we estimated the sequence divergence of full-length TEs, following a previously published method (Faino et al. 2016). This analysis provided additional information beyond the TE phylogenies (fig. 2*A*–*D*), which were based only on the reverse transcriptase domain that each LTR-retrotransposon contains. For each TE, a consensus sequence was generated by aligning all copies of the family across all lineages. Then, the divergence of each copy from the consensus was determined and corrected by the Jukes–Cantor formula (Jukes and Cantor 1969). The same procedure was then repeated with a separate consensus for each lineage. The first method (one consensus for all lineages) showed the distance of each TE from the supposed common ancestor of that TE in all lineages and indicated whether elements in each genome might have proliferated from the same original copy (fig. 4*E*–*H*). The second method (separate consensus for each lineage) showed how diverged the copies within one lineage were and provided information on the recency of each expansion and the population structure of TEs that contributed to it (fig. 4*I*–*L*). The Jukes–Cantor distances in combination with the LTR divergence allowed us to draw hypotheses about the history of each TE family in each lineage (fig. 4*M*–*P*). For *Grasshopper*, we found that the Jukes–Cantor distance metrics could have suggested two expansions of this family, one older and one more recent (fig. 4*G*). However, when taking into account our LTR divergence analysis (fig. 4*C*), it was more likely that the entire *Grasshopper* expansion occurred recently and consisted of multiple copies expanding in parallel (fig. 4*O*). We also looked at where the TE proliferations were localized in the genome and found that the *Grasshopper* expansion occurred globally regardless of the location of the original proliferating copy, as elements from both Jukes–Cantor peaks were distributed throughout MZ5-1-6's seven chromosomes (supplementary fig. S9*A*, Supplementary Material online). In contrast to *Grasshopper*, *MAG_Ty3* appeared to have expanded from a single element only (fig. 4*E*) but also very recently (fig. 4*A*). Our analyses of the *Copia* LTR-retrotransposon revealed a more complex scenario. Firstly, the family of *Copia* elements in MoO and MoS appeared to have proliferated from the same original copy, because most were about the same distance from the consensus of both lineages (fig. 4*J*). MoS *Copia_elem* copies were more similar to each other than those within MoO (fig. 4*F*). Yet, most MoO *Copia* had zero LTR divergence, whereas many MoS *Copia* had further diverged LTRs (fig. 4*B*). The most likely explanation is that the *Copia* expansion in MoO occurred very recently but consisted of multiple elements from an older *Copia* population with sequence differences. Meanwhile, the expansion in MoS was older and caused by just one copy proliferating (fig. 4*N*). As previously mentioned, CRI was skewed above zero for *Copia_elem* in MoS, also suggesting expansion over a longer time period. Finally, *MGRL3* looked to have proliferated from the same original copy in all lineages (fig. 4*L*), which was consistent with the data supporting an old expansion of this family before the lineages diverged (fig. 2*D* and *E*). Overall, we have demonstrated that LTR-retrotransposons in *M. oryzae* have experienced complex proliferation dynamics, resulting in different histories of each lineage-specific expansion.

### A DNA Transposon, *POT2*, Appears to Have Been Transferred From the Rice Pathotype of *M. oryzae* to the Wheat Pathotype

**Fig. 5.**
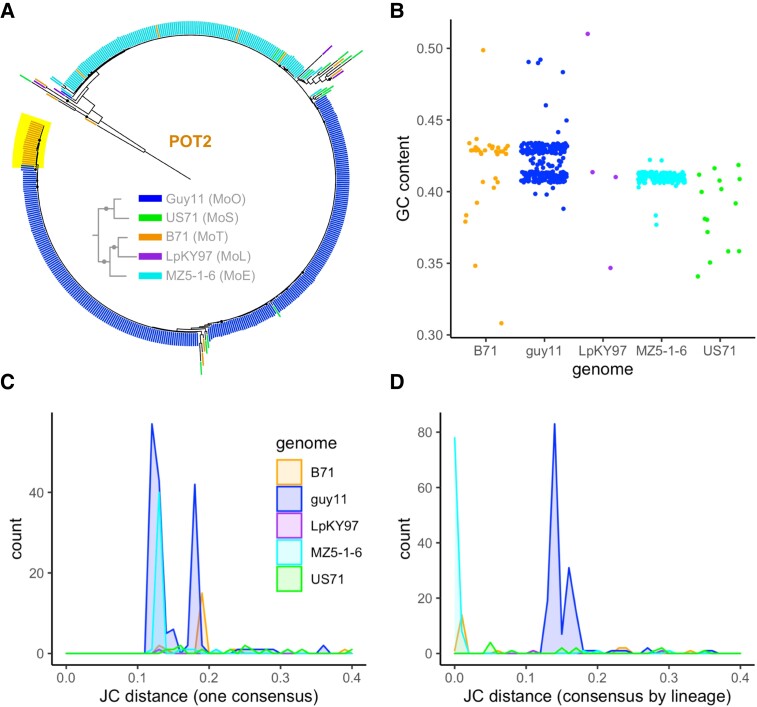
*POT2* was likely transferred between MoO and MoT. (*A*) Domain-based ML phylogeny of *POT2* in each representative genome (Guy11, US71, B71, LpKY97, MZ5-1-6). The yellow highlighted MoT elements indicate those that were potentially transferred, and black circles indicate bootstrap value ≥80. The smaller phylogeny within shows the expected relationships between the lineages for comparison (same as fig. 2*A*) and the color-coding representing the lineage an element is from. (*B*) Jitter plot showing GC content in each *POT2* copy, in each genome. (*C*) Jukes–Cantor distance analysis of *POT2* based on one consensus for all lineages. (*D*) Jukes–Cantor distance analysis of *POT2* based on a separate consensus for each lineage.

Although LTR-retrotransposons had a greater role in increasing the TE content of MoO and MoS, the *POT2* DNA transposon also stood out as being a large contributor (fig. 1). As shown by the ML phylogeny based on alignment of *POT2's* transposase domain, we found it to have greatly expanded in MoO and MoE, with a smaller expansion in MoT (fig. 5*A*). The phylogeny also suggested a potential transfer of *POT2* between MoO and MoT due to the unexpectedly high similarity of certain copies from MoT B71 and MoO Guy11. Previously published criteria for identifying potential TE horizontal transfers (HTs) based on phylogenies are 1) unexpectedly high similarity between TEs in lineages that are not closely related; 2) a patchy distribution of the element in one of those lineages, as well as absence from its sister lineage; and 3) discordance between the TE tree and genome tree (Bergman 2018). When comparing the *POT2* phylogeny with the genome tree based on SCOs (fig. 2*A*), we observed clear discordance between the two. We expected MoT *POT2* to be more closely related to MoE *POT2*, because MoT is closer to MoE than to MoO, but this was not the case. Additionally, *POT2* copies from another MoT genome (BR32) were not found in the clade containing potentially transferred *POT2* from B71 (supplementary fig. S10, Supplementary Material online). BR32 *POT2* copies were also not found to be expanded, likely indicating a patchy distribution of *POT2* in MoT. Finally, *POT2* was generally absent from MoL, the most closely related lineage to MoT. Although there were some older copies of *POT2* in all genomes, including MoL, no *POT2* from MoL were found in or near the clade containing potentially transferred MoT *POT2* (fig. 5*A*). These results are in line with the criteria for a HT of *POT2*.

We also observed additional lines of evidence pointing to a potential transfer of *POT2* between MoO and MoT. Our analysis of GC content revealed that the MoO Guy11 genome had two distinct groupings of *POT2* resembling subfamilies, one with higher GC content and one with slightly lower GC content (fig. 5*B*). Many *POT2* in MoT had the same higher GC content as the former subfamily, and most *POT2* in MoE had the same lower GC content as the latter. The difference in GC content between the *POT2* subfamilies could have been caused by the ancestor of the MoO-MoE *POT2* being slightly RIPped, whereas the MoO-MoT *POT2* ancestor had not, then each element having had its own evolutionary trajectory thereafter. The Jukes–Cantor analysis further supported the trend observed from the GC content analysis. When comparing *POT2* copies from all lineages with their consensus, we saw the same two MoO-MoT and MoO-MoE subfamily groupings (fig. 5*C*). This supported the idea that the two groups did not come from the same original *POT2* element. Instead, they likely originated from separate elements with sequence differences. Although the MoO-MoE grouping of *POT2* by GC content and Jukes–Cantor distance might resemble a transfer between these lineages as well, it is not supported by the phylogeny (fig. 5*A*). Additionally, *POT2* in MZ5-1-6 had a high CRI value of almost 2 whereas *POT2* in Guy11 and B71 did not (supplementary fig. S7, Supplementary Material online), which suggested that the expansion in MZ5-1-6 may have been caused by an ancestral *POT2* copy, going against the idea of a HT. Thus, the original MoO-MoE *POT2* was likely present in all lineages but only expanded in MoO and MoE, whereas the MoO-MoT *POT2* expanded only in MoO then transferred to MoT. Comparing each *POT2* copy to the consensus of its lineage (fig. 5*D*) showed that *POT2* in MoT and MoE were more closely related within their respective lineages than *POT2* within MoO. This suggested that either *POT2* expansions in MoT and MoE occurred much more recently than in MoO or that they consisted of a single element expanding, whereas multiple elements from a population of TEs with sequence differences expanded in MoO. Either interpretation supported the idea that an individual *POT2* was recently transferred from MoO to MoT and subsequently expanded.

*POT2* also experienced differential localization of its expansions in different lineages. Local proliferation was displayed by *POT2* in MoT, where most of its copies were located on the minichromosome sequences of the B71 genome (supplementary fig. S9*B*, Supplementary Material online). In contrast, *POT2* in MoE was evenly distributed throughout the seven chromosomes (supplementary fig. S9*C*, Supplementary Material online), which was similar to the LTR-retrotransposon expansions we characterized. Minichromosomes have been reported to harbor many repetitive sequences as well as virulence factors (Langner et al. 2021). Of the genomes used in this study, it is known that MZ5-1-6 (MoE), BR32 (MoT), and Guy11 (MoO) do not have minichromosomes, whereas B71 (MoT), LpKY97 (MoL), FR13 (MoO), US71 (MoS), and CD156 (MoE) do (Peng et al. 2019; Langner et al. 2021). Despite the existence of genomes both with and without minichromosomes in many of the lineages, their presence did not affect the lineage-specific patterns of TE content (fig. 1). Since the MoE MZ5-1-6 genome does not contain minichromosomes and experienced a global *POT2* expansion, whereas B71 does have minichromosomes and had a local *POT2* expansion there, it is possible that the presence of minichromosomes affects the overall localization of TE content in *M. oryzae*. Regardless, this result further highlights the different expansion histories and dynamics of *POT2* in different lineages.

### *POT2* May Have Been Transferred During a Recombination Event Between Isolates of the Rice and Wheat *M. oryzae* Pathotypes

**Fig. 6.**
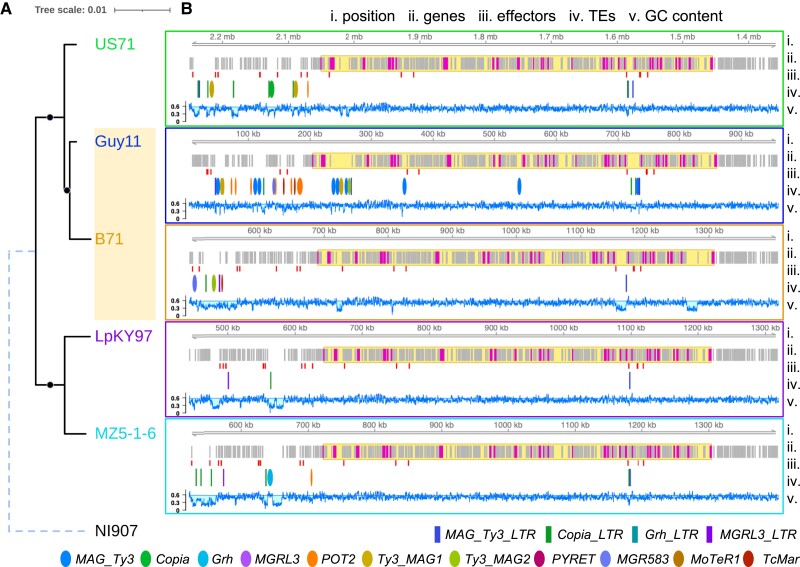
A large region may have been transferred between MoO and MoT isolates as a result of recombination. (*A***)** The ML phylogeny of an ∼583 kb syntenic region on chromosome 7 that contains many genes following a *POT2*-like gene tree topology. Black circles indicate a bootstrap value of 100. Branch lengths are to scale, except for the dashed outgroup branch of NI907 (*M. grisea*). (*B***)** Genomic tracks show features of the potential region of recombination in each genome. Tracks from top to bottom: i) position along the scaffold or chromosome (B71: CM015706.1, Guy11: MQOP01000008.1, US71: UCNY03000007.1, LpKY97: CP050926.1, MZ5-1-6: CP034210.1, NI907: CM015044.1). ii) All genes, where magenta represents a *POT2*-like topology gene, and the yellow highlighted area indicates the region containing all of those genes. iii) Position of candidate effectors. iv) Position of TEs, where ellipses are full elements (blue, *MAG_Ty3*; green, *Copia_elem*; teal, *Grasshopper*; purple, *MGRL3*; orange, *POT2*; mustard, *Ty3_MAG1*; yellow green, *Ty3_MAG2*; magenta, *PYRET*; sky blue, *MGR583*; light brown, *MoTeR1*; tomato, *TcMar_elem*), and rectangles are solo-LTRs (dark blue, *MAG_Ty3_LTR*; dark green, *Copia_LTR*; dark teal, *Grasshopper_LTR*; dark purple, *MGRL3_LTR*). v) GC content, where the horizontal line is the genome-wide average GC content of coding regions (0.577891).

To investigate how *POT2* may have been transferred between the MoO and MoT lineage, we first looked for any regions of the Guy11 and B71 genomes that may have been involved in a larger transfer event. *POT2* elements and their flanking regions were compared using DNA alignments and synteny analysis; however, none of these segments containing *POT2* stood out as being potentially transferred regions. We then considered the possibility that *POT2* could have moved as part of a larger region but then transposed out of that region. We looked for evidence of genes that might have been transferred between Guy11 and B71 by filtering for gene trees that followed the same topology as the *POT2* phylogeny. One region in B71 on chromosome 7 stood out as having many of these genes, with 29 out of the 38 genes that matched the *POT2* phylogeny being located there (supplementary fig. S11*A*, Supplementary Material online). The other 9 genes were scattered among various chromosomes. We located the orthologs of the 29 B71 genes in the other genomes and found them to be syntenic. In LpKY97 and MZ5-1-6, the other two chromosome level assemblies, the genes were in the same location on chromosome 7. We aligned the full-length nucleotide sequence from each genome and produced an ML phylogeny (fig. 6*A*), which showed that this entire region followed a *POT2*-like tree topology rather than the expected evolutionary relationships between the *M. oryzae* lineages (fig. 2*A*). Since B71 grouped with Guy11 in the MoO-MoS clade, it was likely that an MoO isolate was the donor of this region in B71. Additionally, because this region was syntenic in all genomes, the most likely explanation for the transfer event was homologous recombination.

We then looked at the TE insertions in the region we identified to determine the timing of the TE expansions we characterized in relation to the transfer of the region. There were no full-length TEs contained in this region besides in the Guy11 genome, where *MAG_Ty3*, *Ty3_MAG1*, and *Ty3_MAG2* elements were likely inserted after the transfer event. There were a few solo-LTRs located in the region, including a *MAG_Ty3* solo-LTR that was present at the same location in all genomes. Located upstream of the transferred region, there was a unique set of many TEs in each genome, indicating lineage-specific TE activity. There were no *POT2* within or nearby this region in B71; however, there were many *POT2* copies upstream of the region in Guy11 (fig. 6*B*), supporting the possibility that one of these elements were included in the recombination event.

Finally, we investigated if any genes of importance were transferred along with this region. There were a few predicted effectors (fig. 6*B*); however, they were not under presence–absence variation and did not include any AVRs or members of expanded *M. oryzae* ART and MAX effector families (Seong and Krasileva 2021). We then characterized the genes in this region by obtaining their Gene Ontology (GO) terms (Additional File 3) and PFAM domain terms (Additional File 4). The most common terms included a putative ssRNA binding PFAM domain (RRM_1), iron ion binding molecular function (MF), zinc ion binding MF, proteolysis biological process (BP), glycolytic process BP, DNA repair BP, and mitochondrion cellular component (CC). Although the region was too small (182 genes out of 12,658 total) to perform meaningful enrichment analysis, it notably had similar characteristics to a recently discovered group of large mobile elements known as *Starships*, due to the presence of effectors, metal-binding genes, and other conserved genes (Gluck-Thaler et al. 2022). Within the region, there were genes containing NOD-like receptor (NLR)-associated domains (HET, Ank_2, Ank_4, Ank_5) and ferric reductase-associated domains (FAD_binding_4, FAD_binding_7, NAD_binding_2, NAD_binding_11), both of which are conserved genes in *Starships* (Gluck-Thaler et al. 2022). Although tyrosine recombinase (DUF3435) and patatin-like phosphatase *Starship*-associated domains were not present within the region, a nearby upstream gene in B71 contained a fragmented DUF3435 (supplementary fig. S11*B*, Supplementary Material online). It is possible that this region could have originated from a *Starship* element; however, it would likely be much older than lineage divergence, given the synteny in all genomes. Thus, our results still strongly suggest that recombination caused the transfer of the region. Yet, it is interesting to consider the potential origins of the region, due to the adaptive function often conferred by mobile *Starship* elements (Gluck-Thaler et al. 2022).

## Discussion

Differences in TE content contribute to intraspecific diversity in many fungal species (Castanera et al. 2016; Shirke et al. 2016; Chen et al. 2018; Bleykasten-Grosshans et al. 2021; Oggenfuss et al. 2021; Gourlie et al. 2022). To understand how TE content variation arises and may relate to the evolutionary history of fungal pathogens, we constructed a de novo TE library that represents the diversity of TEs in five *M. oryzae* lineages. Using this library, we found that MoO and MoS contain much greater TE content than MoT, MoL, and MoE. Our pipeline ensured that these differences were not due to any bias of the TE library toward particular lineages, and correlation tests showed that they were not due to differences in genome assembly or quality. Although we focused on elements containing TE-associated domains (Muszewska et al. 2019), further work is needed to study the full set of all repetitive DNA across the *M. oryzae* lineages. Most notably, protein domain-lacking elements such as SINEs and MITEs are not included here (supplementary table S2, Supplementary Material online), so our analyses likely underestimate overall TE content. Additionally, our analysis was restricted to highly contiguous genome assemblies which are few in number for *M. oryzae*, especially for the MoS, MoL, and MoE lineages. Although we are confident that our analyses are robust for the TE families we characterized, we may have missed other isolate-specific TE expansion events.

Despite these limitations, we found strong evidence that recent lineage-specific TE expansions contributed to the greater number of TEs in MoO and MoS. Analyzing solo-LTR copy numbers allowed us to verify that some LTR-retrotransposons were expanded in certain lineages, rather than having been expanded in all lineages and subsequently removed in only some. By synthesizing the results of our LTR divergence and Jukes–Cantor distance analyses, we were able to construct a model showing differences in the method of TE expansion between various types of TEs and between the same TE in different lineages. Some expansions were caused by the proliferation of one element, whereas others consisted of multiple elements from an older population of TEs with sequence differences proliferating in parallel. Most expansions occurred globally, with elements being distributed throughout the genome; however, the *POT2* DNA transposon proliferated locally in the MoT isolate (B71) minichromosomes. Solo-LTR and LTR divergence analyses are not possible for DNA transposons, so it is difficult to determine expansion versus contraction dynamics for *POT2* and how recently its copies proliferated. Nevertheless, our reconstruction of TE expansion histories points to the complexity of TE activity in *M. oryzae*.

Through our analyses, we found multiple lines of evidence suggesting the recent transfer of a DNA transposon between rice- and wheat-infecting *M. oryzae* lineages. The phylogeny, Jukes–Cantor distances, and GC contents of *POT2* copies all showed that MoT *POT2* grouped unexpectedly with MoO *POT2* when considering the evolutionary relationships between the lineages. Given that *POT2* has been found to insert into the *AVR-Pib* effector gene in MoO field isolates and modulate their virulence (Li et al. 2023), its transfer to other lineages has the potential to contribute to their adaptability. The potential transfer of *POT2* could have occurred by a variety of mechanisms, including HT or recombination between the lineages. Notably, *POT2* is a DDE-type DNA transposon of the Tc1/Mariner family, which are reported to be prone to HT (Wells and Feschotte 2020). However, we could not identify direct evidence of such an HT event. It is possible that an individual *POT2* transferred by itself, which would not be possible to detect through a comparative genomics approach, given that DNA transposons leave almost undetectable excision footprints (Luo et al. 1998). Additionally, we did not find evidence of any nonsyntenic, horizontally transferred regions that could have carried *POT2*. Since the potentially transferred *POT2* copies are localized on B71's minichromosomes, it is also possible that minichromosome dynamics allowed *POT2's* transfer or resulted in its localization. The HT of minichromosomes between isolates has been previously observed (Langner et al. 2021), as has the acquisition of core chromosomal regions by minichromosomes (Peng et al. 2019). An alternative explanation for the transfer of *POT2* is gene flow between the lineages through recombination during sexual reproduction. A previous study has shown evidence of historical gene flow in *M. oryzae*, most of which was caused by events that occurred before the divergence of the lineages (Gladieux, Condon, et al. 2018). However, the hypothesis that MoT and MoL arose within the last 60 years via a large admixture event involving recombination between isolates of various pathotypes in South America (Rahnama et al. 2021) makes it possible that *POT2* was acquired by MoT in that event.

**Fig. 7.**
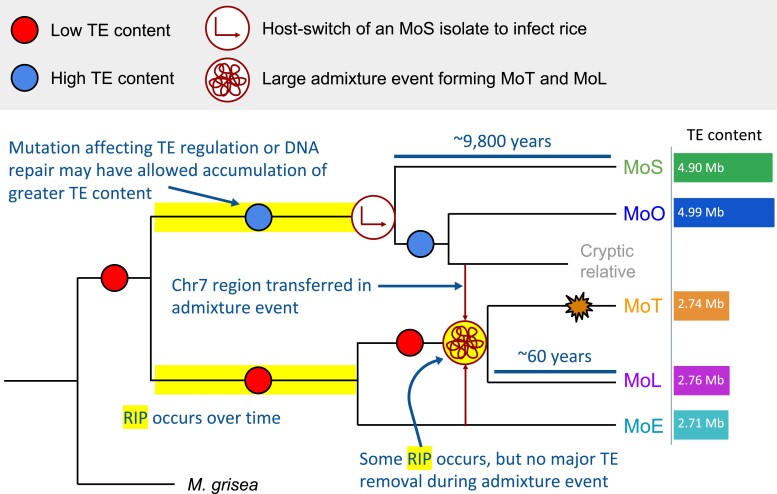
Proposed model showing how the MoO-MoS and MoT-MoL-MoE lineage groups have likely experienced different evolutionary histories. MoO-MoS divergence via host switch is well accepted, whereas our results indicate that MoT and MoL were formed in a large admixture event, rather than as a result of a conventional host switch. The diversity present in MoO-MoS accumulated over ∼9,800 years, whereas the diversity in MoT-MoL was generated over the past 60 years. Our results suggest that a lower TE content was likely the ancestral state of the *M. oryzae* lineages. We hypothesize that some difference, possibly a mutation affecting a TE regulation or DNA repair pathway, may have contributed to the increased TE content in MoO and MoS. The total length of TEs for the representative genome of each lineage is shown at the right of the tree to highlight the difference in TE content. RIP (highlighted in yellow) may have occurred during the large admixture event forming MoT and MoL but is not sufficient to explain the higher TE content in MoO and MoS. During the admixture event, a region on chromosome 7 (Chr7) was transferred from a cryptic relative of the MoO lineage and may have contained a *POT2* element, which subsequently expanded in the MoT lineage (orange starburst).

In searching for genes that might have accompanied *POT2* in a potential transfer event, we identified a region of recombination between MoO and MoT. This region on chromosome 7 contained many genes whose phylogenies followed the topology of the *POT2* phylogeny and were syntenic in each lineage. Although our analyses do not rule out the possibility of incomplete lineage sorting, this region was also identified by Rahnama et al. as originating from a currently unsampled (cryptic) relative of MoO and MoS (Rahnama et al. 2021) that participated in recombination during the large admixture event. This likely occurred before the divergence of MoT and MoL, because a few MoL isolates appear to contain the region and a few MoT isolates do not (Rahnama et al. 2021). This strongly suggests that the region we identified experienced recombination. The TE insertions in this region in the MoO isolate provide further evidence that the transferred region originates from a relative of MoO that participated in the admixture event, because it is unlikely that this region accumulated eight new LTR-retrotransposon insertions in the past 60 years (fig. 6*B*). Since we did not find a copy of *POT2* in the transferred region, there was no direct evidence that POT2 transferred by this mechanism. However, the exact boundaries of the transferred region are unclear, so it is possible that *POT2* copies present upstream in MoO Guy11 could have been included. Thus, we highlight this region as an example of a potential way that *POT2* may have been transferred. We propose that *POT2* was transferred from the cryptic relative of MoO and MoS to an ancestor of MoT and MoL in the recombination event involving this region. Subsequently, *POT2* could have transposed out of the transferred region to B71's minichromosomes, where it proliferated (fig. 7).

Taken together with previous findings, our results suggest that the MoO-MoS and MoT-MoL-MoE lineage groups have experienced distinct evolutionary histories. MoO and MoS are set apart by their greater TE content; although the TE expansions we characterized help to explain some of the difference, it is still unclear how they accumulated approximately twice as many TEs as the other lineages. We hypothesize that low TE content was the ancestral state of *M. oryzae* before lineage divergence and perhaps a mutation causing a difference in a TE regulation or DNA repair mechanism in the ancestor of MoO-MoS allowed the accumulation of greater TE content over time (fig. 7). Genes involved in DNA repair are of particular interest due to the recent finding that multiple noncanonical and error-prone DNA repair pathways exist in *M. oryzae* and their influence on genomic variation are not well understood (Huang et al. 2022). Notably, TEs in *M. oryzae* have been found to activate in response to stress (Chadha and Sharma 2014), so various environmental and host–plant conditions may have also played a role in TE content differences. MoT, MoL, and MoE are distinguished by their greater isolate-specific TE content variation (fig. 1), and multiple lines of evidence suggest that MoT and MoL were the result of a large admixture event (Rahnama et al. 2021), rather than a host switch of MoL to infect wheat as previously hypothesized (Inoue et al. 2017). This is in contrast to MoO and MoS, which are well established to have diverged via a host switch of MoS to rice (Couch et al. 2005; Gladieux, Ravel, et al. 2018; Zhong et al. 2018). The two hypotheses represent quite different evolutionary processes, as a host switch implies steady adaptation and selection for mutations that allow infection of a new host, whereas a large admixture event may quickly bypass the process of adaptation by generating lots of diversity, causing some strains to infect new hosts. Along with the argument presented by Rahnama et al. (Rahnama et al. 2021), our findings of the potentially transferred *POT2* and region of recombination align with the admixture hypothesis for MoT and MoL. Finally, although the diversity represented in the genome tree by branch lengths is comparable for the two lineage groups (supplementary fig. S2, Supplementary Material online), it is important to note that the diversity we observed was likely generated much more rapidly for MoT-MoL in the last 60 years (Rahnama et al. 2021), compared with MoO-MoS, where it seems to have accumulated steadily over 9,800 years (Gladieux, Ravel, et al. 2018). A large admixture event recently forming MoT and MoL, as compared with the host switch resulting in the divergence of MoO and MoS, would explain these differences well.

In formulating our model (fig. 7), we considered whether the large admixture event presented by Rahnama et al. (Rahnama et al. 2021) might explain the low TE content of MoT and MoL, where many TEs could have been mutated beyond recognition by RIP and removed via recombination during the event. However, our data supports the alternative hypothesis that all lineages originally had lower TE content and MoO-MoS independently accumulated their greater TE content after divergence from the common ancestor of all lineages (fig. 7). MoE genomes have low TE content, so this is not unique to the MoT-MoL isolates that originated from the admixture event. Likewise, the fact that TE content of the *M. grisea* outgroup is most similar to MoT-MoL-MoE supports the idea that the common ancestor of all lineages had low TE content. Additionally, if many TEs were removed during the admixture event, we would expect large numbers of solo-LTRs in MoT and MoL, because recombination between flanking LTRs is a common mechanism of removal. However, our results indicate no extensive removal in MoT and MoL to explain the large difference in TE content. A lack of severe RIP in the TEs we analyzed also refutes the idea that RIP mutated TEs beyond recognition in the genomes of all lineages except MoO and MoS. Through our GC content analysis (fig. 3, supplementary fig. S8, Supplementary Material online), we concluded that there had not been recent RIP affecting TEs in any *M. oryzae* lineages. It is very likely that RIP was active during the admixture event because there were many sexual recombinations, and Rahnama et al. found regions that had clearly been RIPped (Rahnama et al. 2021). However, this RIP activity was not sufficient to explain the differences in TE content we observed. The strongest evidence showing that RIP had not severely affected TEs during the admixture event is that the *MGRL3* LTR-retrotransposon, which we found to be an old element that proliferated before the divergence of the lineages, had not experienced recent RIP in any of the lineages (fig. 3). If RIP affected *MGRL3* during the admixture, we would expect to find less copies in MoT and MoL compared with other lineages. However, its copy number of full elements and solo-LTRs was very uniform throughout all lineages. Thus, we propose that there is a biological difference between the MoO-MoS and MoT-MoL-MoE clades responsible for the drastic difference in TE content, likely related to their different evolutionary histories (fig. 7).

In this study, we have shown that lineage-specific TE content differences in *M. oryzae* were caused in part by complex lineage-specific TE expansion dynamics. Future studies might investigate potential DNA repair or TE regulation mechanisms to better understand how the large differences between the MoO-MoS and MoT-MoL-MoE lineage groups arose. Additionally, the dynamics of a DNA transposon potentially transferred from MoO to MoT led us to identify a region of recombination between the two lineages. Our results support the hypothesis that the MoT and MoL lineages were formed in a large admixture event rather than caused by a host switch, suggesting that their evolutionary history was vastly different from MoO and MoS. Further work is needed to investigate the consequences of differing evolutionary histories on the adaptive potential and trajectory of *M. oryzae* lineages. This study demonstrates that investigating TE dynamics can help us to better understand intraspecific diversity, which is especially important in fungal pathogens with host-specific populations.

## Materials and methods

### Genomic Data Sets Used and Quality Assessment

All genome sequences were retrieved from NCBI GenBank in December 2020, along with information on the host they were isolated from, the year they were collected, their GenBank accession, the assembly quality, the number of scaffolds, and the genome size (supplementary table S1, Supplementary Material online). Isolates were chosen primarily based on having the lowest number of contiguous scaffolds, which is ideal for TE annotation (Rech et al. 2022). We assessed the completeness of the genomes using BUSCO (Manni et al. 2021) version 5.2.2 software with “sordariomycetes_odb10” as the busco_dataset option.

### TE Annotation, Classification, and Phylogenetic Analysis

The highest quality representative genomes of each lineage (Guy11 for MoO, US71 for MoS, B71 for MoT, LpKY97 for MoL, and MZ5-1-6 for MoE) were used as input into Inverted Repeat Finder (Warburton et al. 2004) version 3.07 and RepeatModeler (Flynn et al. 2020) version 2.0.2 software to obtain de novo annotations of TEs representing all lineages. Inverted Repeat Finder was called with options “2 3 5 80 10 20 500000 10000 -a3 -t4 1000 -t5 5000 -h -d -ngs”, and RepeatModeler was called with options “-engine ncbi -LTRStruct”. These libraries were combined with the RepBase (Bao et al. 2015) fngrep version 25.10 library of known TEs in fungi, and clustering was performed using cd-hit-est from CD-HIT (Fu et al. 2012) version 4.7 with options “-c 1.0 -aS 0.99 -g 1 -d 0 -M 0”. The resulting comprehensive TE library was then scanned against a list of domains containing TE-coding sequences (Muszewska et al. 2019), consisting of both CDD profiles and PFAM profiles. The TE library was scanned for CDD profiles using rpsblastn from ​​Blast (NCBI Resource Coordinators et al. 2018) version 2.7.1+ with option “-evalue 0.001”. PFAM profiles were retrieved from the Pfam-A.hmm file included with HMMER (Eddy 2011) version 3.1b2. PFAM hmms were used for domain scanning along with pfam_scan.pl (Finn et al. 2016) version 1.6 with options “-e_dom 0.01 -e_seq 0.01 -translate all”. Elements not containing at least one of either a PFAM or CDD domain were filtered out.

Next, new sequences generated from de novo repeat-finding were classified in the library. Domain-based ML phylogenies were constructed using the most common PFAM domains found in the TE library, which were RVT_1 (PF00078.29), DDE_1 (PF03184.21), rve (PF00665.28), Chromo (PF00385.26), RNase_H (PF00075.26), and RVT_2 (PF07727.16). Each domain was aligned to the TE library using HMMER hmmalign (Eddy 2011) version 3.1b2 with options “--trim --amino --informat fasta”. The alignments were processed using esl-reformat and esl-alimanip from Easel version 0.48, which is part of the HMMER (Eddy 2011) package. Columns containing all gaps were removed by calling esl-reformat with the “--mingap” option, so that the length of the alignment was the same as the hmm length. Then, sequences that did not match at least 70% of the hmm were filtered out by calling esl-alimanip with the “--lmin” option specifying 70% of the hmm length. Finally, esl-reformat was used to convert the alignment to fasta format. RAxML (Stamatakis 2014) version 8.2.11 with options “-f a -x 12345 -p 12345 -# 100 -m PROTCATJTT” was then used to construct the domain-based ML phylogeny of TEs containing the domain. This process was repeated for each of the six domains. The phylogenies were visualized in the Interactive Tree of Life (iTOL) (Letunic and Bork 2019) online tool, and clades where de novo elements grouped with elements having a classification in the RepBase library were copied using the “copy leaf labels” feature of iTOL. *De novo* elements in the clade were then classified as being part of the same family as the known element from RepBase (supplementary fig. S1, Supplementary Material online), generating a TE library with many more elements having classifications.

This classified TE library and the full set of genomes were then used as input to RepeatMasker (Smit et al. 2013) version 4.1.1 with options “-gff -cutoff 200 -no_is -nolow -gccalc” to generate copy number and positional data for TEs in all of the genomes. These hits were converted to fasta format using bedtools (Quinlan and Hall 2010) getfasta version 2.28.0 with the “-s” option to force strandedness and then filtered once more for elements containing a TE-coding sequence domain, as described previously. This produced TE annotations for each genome of elements that were predicted to be complete.

Data from the TE annotations on copy number of each TE family, total length each TE family occupies, and percentage of TE content were used for PCA, calculated using the prcomp (R Core Team) function and visualized with ggbiplot (Vu 2011) version 0.55 in R version 4.1.0. Point-biserial correlation coefficients were calculated using the cor (R Core Team) function in R version 4.1.0 between binary (LineageGroup) and continuous (all other) variables from supplementary table S1, Supplementary Material online, and Spearman correlation coefficients were calculated between all pairs of continuous variables. For the binary LineageGroup variable, MoO and MoS genomes were assigned a value of 1, and MoT, MoL, and MoE genomes were assigned a value of 0.

Domain-based ML phylogenies of each TE family were constructed in the same way as those used to give de novo elements a family classification. The domains (with Pfam accession) used for each TE were RVT_1 reverse transcriptase (PF00078.29) for *MAG_Ty3*; *Grasshopper*, *Ty3_MAG1*, *MGR583*, *PYRET*, *MoTeR*, and RVT_2 reverse transcriptase (PF07727.16) for *Copia_elem*; rve integrase (PF00665.28) for *MGRL3* and *Ty3_MAG2*; and DDE_1 transposase (PF03184.21) for *POT2* and *TcMar_elem*.

### Phylogeny of *M. oryzae* Genomes

The genome tree of the *M. oryzae* isolates was generated by first annotating genes in each genome using FunGAP (Min et al. 2017) version 1.1.0 with arguments “--augustus_species magnaporthe_grisea --busco_dataset sordariomycetes_odb10”. RNAseq data for genome annotation was retrieved from the NCBI Sequence Read Archive (SRA) database in June 2021. RNAseq for Guy11 (accession SRX5630771) was used as input for genomes of MoO and MoS lineage, RNAseq for B71 (accession SRX5900622) was used for MoT and MoL genomes, and RNAseq for MZ5-1-6 (accession SRX5092987) was used for MoE genomes. This resulted in predicted genes for each genome, which were input to OrthoFinder (Emms and Kelly 2019) version 2.5.4 along with the *M. grisea* NI907 proteome as the outgroup (retrieved from NCBI GenBank, accession GCA_004355905.1). OrthoFinder was run with options “-M msa -S diamond_ultra_sens -A mafft -T fasttree”, and the output identified 8,655 SCOs. These were aligned using MAFFT (Katoh and Standley 2013) version 7.312 with parameters “--maxiterate 1000 --globalpair”, and the alignments were concatenated. The ML phylogeny was produced from the alignment using RaxML (Stamatakis 2014) version 8.2.11 with options “-m PROTGAMMAGTR -T 24 -f a -x 12345 -p 12345 -# 100” and was visualized in iTOL (Letunic and Bork 2019).

### Divergence Analysis

To characterize RIP in *M. oryzae*, GC content was calculated using geecee from EMBOSS (Rice et al. 2000) version 6.6.0.0 in TEs and in coding sequences of the representative genomes. The CRI per element was also determined using The RIPper tool (Van Wyk et al. 2019). Median and median absolute deviation (MAD) values were calculated for each TE in each genome in R version 4.1.0 using the med and mad functions (R Core Team).

LTR divergence analysis was performed by first determining a consensus sequence for each flanking LTR. Elements from the *MAG_Ty3*, *Copia_elem*, *Grasshopper*, and *MGRL3* domain-based phylogenies were extracted from each representative genome, plus 1,000 bp on either side, using bedtools slop (Quinlan and Hall 2010) version 2.28.0. These sequences were then aligned using BlastN from Blast (NCBI Resource Coordinators et al. 2018) version 2.7.1+ against the clustered TE library from the intermediate step in the TE annotation pipeline, before LTRs were removed when filtering for domain-containing elements (supplementary fig. S1, Supplementary Material online). This helped to manually determine the element that best represented the LTR sequence of each TE, which was aligned, again using BlastN (NCBI Resource Coordinators et al. 2018), back to the set of LTR-retrotransposon sequences plus flanking regions to extract LTRs. These extracted LTRs were then aligned using MAFFT (Katoh and Standley 2013) version 7.312, and a consensus sequence was generated using EMBOSS cons (Rice et al. 2000) version 6.6.0.0. The resulting LTR consensus sequences were used as the input library to RepeatMasker (Smit et al. 2013) version 4.1.1 with options “-gff -cutoff 200 -no_is -nolow -gccalc”, which produced positional information for all LTRs. This was used along with the original full sequence plus flanking regions to find which LTRs belonged to which full elements using bedtools intersect (Quinlan and Hall 2010) version 2.28.0. Finally, EMBOSS needle (Rice et al. 2000) version 6.6.0.0 was used to find the divergence of flanking LTR pairs.

Jukes–Cantor distance analysis was performed on all full-length TEs of interest, where the distances of each element to the consensus of its lineage and to the consensus of all copies of that TE from any lineage were calculated. Following previous methods (Faino et al. 2016), we first produced the two types of consensus sequences by aligning TEs using MAFFT (Katoh and Standley 2013) version 7.312 and then using EMBOSS cons (Rice et al. 2000) version 6.6.0.0 to generate the consensus of the alignment. The divergence of a TE from the consensus was found using EMBOSS needle (Rice et al. 2000) version 6.6.0.0, and this divergence was corrected by the Jukes–Cantor distance formula (Jukes and Cantor 1969). Using *Copia_elem* as an example, a consensus for all lineages was generated by aligning all copies of *Copia_elem* present in its domain-based ML phylogeny, and then, the distance of all *Copia_elem* from that consensus was found and plotted separately for each lineage. Also, a consensus was generated separately for *Copia_elem* from MoO, and the distance was computed as previously described, except using this consensus specific to the lineage. This was done for *Copia_elem* copies in each lineage separately and plotted. This process for making both plots was done for each of *MAG_Ty3*, *Grasshopper*, *POT2*, and *MGRL3* as well.

### Solo-LTR Analysis

Solo-LTRs were identified by determining which LTRs (from the annotations previously generated for LTR divergence analysis) did not belong to an LTR-retrotransposon found by the TE annotation pipeline. Using the “-v” option for bedtools intersect (Quinlan and Hall 2010) version 2.28.0 returned only the LTR sequences that had no overlap with an annotated TE and thus were considered solo-LTRs. The number of solo-LTRs compared with the number of their full-length LTR-retrotransposon counterparts within and across genomes was used to determine whether the retrotransposon experienced expansion or removal from the genome.

### Analyses for Investigating Potential *POT2* HT

To investigate potential larger HT regions containing *POT2*, synteny analyses were performed between all *POT2* regions in Guy11 and B71. *POT2* sequences plus 50,000 bp on either side were extracted using bedtools slop and getfasta (Quinlan and Hall 2010) version 2.28.0. These regions were compared using nucmer and mummerplot from MUMmer (Marçais et al. 2018) version 4.0.0. To align the sequences, nucmer was called with the “--maxmatch” option, and to visualize the alignment, mummerplot was called with options “--postscript --color”. This produced synteny plots that were visually screened through for long segments of synteny between Guy11 and B71 flanking the position of *POT2*.

In order to find any genes that may have been transferred along with *POT2*, gene trees produced by OrthoFinder based on amino acid sequence were screened to select those that follow the same topology as the *POT2* phylogeny. The ete2 (Huerta-Cepas et al. 2010) python package version 2.3.10 was used to determine which gene trees were structured such that the gene from Guy11 (MoO) and the gene from B71 (MoT) had the smallest distance from each other than from any other gene. Out of all SCOs, 388 genes had trees following this topology, and these were further refined by aligning their nucleotide sequences and determining topology in the same way as before. The remaining 38 genes whose trees based on nucleotide sequence followed this topology were visualized in IGV (Robinson et al. 2011) to determine any localization in the B71 genome.

### Investigating the Potential Region of Recombination

The full segments of chromosome 7 from each representative genome that contained genes following a *POT2* topology were extracted using bedtools getfasta (Quinlan and Hall 2010) version 2.28.0, and the nucleotide sequences were aligned using MAFFT (Katoh and Standley 2013) version 7.312. A phylogeny was produced using RAxML (Stamatakis 2014) version 8.2.11, with options “-m GTRGAMMA -T 20 -f a -x 12345 -p 12345 -# 100” based on the alignment.

To characterize the genes located in the potential region of recombination, we obtained their GO terms (Additional File 3) using the PANNZER (Törönen et al. 2018) webserver and their PFAM terms (Additional File 4) using pfam_scan.pl (Finn et al. 2016) version 1.6 with options “-e_dom 0.01 -e_seq 0.01” against the Pfam-A.hmm library of HMMER (Eddy 2011) version 3.1b2. The output from PANNZER was then filtered for GO terms with positive predictive value (PPV) value > 0.6.

### Effector Annotation and Analysis

Effectors were predicted by following a previously established pipeline (Singh et al. 2019). Proteomes from FunGAP (Min et al. 2017) output were input into SignalP (Almagro Armenteros et al. 2019) version 5.0 to filter for proteins containing a signal peptide. The output of SignalP was then input to tmhmm (Krogh et al. 2001) version 2.0, which filtered out proteins containing a transmembrane domain. Finally, the remaining proteins were input to EffectorP (Sperschneider and Dodds 2022) version 3.0.

These predicted effectors were then used to investigate their association with TEs, following previous method (Joubert and Krasileva 2022). The 5′ (upstream) and 3′ (downstream) distances of TEs to effectors were found using bedtools (Quinlan and Hall 2010) closest, with options “-D a -id -t first” to find the closest upstream distance by excluding downstream sequences and “-D a -iu -t first” to find the closest downstream distance by excluding upstream sequences. Permutation tests with 10,000 replicates were then performed for the 5′ distance, 3′ distance, and minimum distance on either side to test the significance of the median distance of each TE family to effectors, compared with the median distance of all other TEs to effectors in each genome. The same distance analyses and permutation tests were also performed on ART and MAX effectors (Seong and Krasileva 2021) versus non-ART and non-MAX effectors.

### Data Processing and Analysis

Analyses were conducted in a Linux environment with GNU bash version 4.2.46, GNU coreutils version 8.22, GNU Awk version 4.0.2, GNU grep version 2.20, and gzip version 1.5. Conda version 4.10.1 was used to install software. Scripts for parsing data were written in Python version 3.7.4, using biopython (Cock et al. 2009) version 1.79. R (R Core Team) version 4.1.0 was used to write scripts for data analysis and plotting, with packages ggplot2 (Wickham 2016) version 3.4.1, RColorBrewer (Neuwirth 2022) version 1.1-3, dplyr (Wickham et al. 2023) version 1.1.0, tidyverse (Wickham et al. 2019) version 2.0.0, and scales (Wickham and Seidel 2022) version 1.2.1.

## Supplementary Material

evad206_Supplementary_Data

## Data Availability

Data sets and intermediate analyses files are provided as additional data files or available on Zenodo at DOI 10.5281/zenodo.7366416. Code and scripts used for all analyses are located in a GitHub repository (https://github.com/annenakamoto/moryzae_tes).
