# Supplementary material for: Intraspecific Variation of Transposable Elements Reveals Differences in the Evolutionary History of Fungal Phytopathogen Pathotypes
Source: Genome Biol Evol. 2023 Nov 17;15(12):evad206. doi: 10.1093/gbe/evad206 (PMC10691877; doi:10.1093/gbe/evad206)
Supplement: evad206_Supplementary_Data [file evad206_supplementary_data.zip › moryzae_tes_V3_supplemental_revision.docx]

**Additional File 1: Supplementary Figures and Tables.**

**Table S1:** *M. oryzae* genomes used in this study, with the lineage they belong to, host they were isolated from, collection year, NCBI accession, assembly level, number of scaffolds, percentage of complete BUSCOs, genome size in base pairs, and the percentage of the genome containing TEs (as found by our TE annotation pipeline). The highest quality representative genome used for each lineage is highlighted in yellow. All genomes were retrieved from NCBI GenBank December 2020.

| **Isolate** | **Lineage** | **Host** | **Year** | **NCBI accession** | **Assembly Quality** | **# Scaffolds** | **N50** | **L50** | **% Complete BUSCOs** | **Genome size (bp)** | **% TEs in genome** |
| --- | --- | --- | --- | --- | --- | --- | --- | --- | --- | --- | --- |
| AG006 | *Oryza* | *Oryza sativa* | 2011 | GCA_905067025.2 | Contig | 24 | 6,618,557 | 3 | 95 | 47,005,811 | 11.41 |
| AG039 | *Oryza* | *Oryza sativa* | 2011 | GCA_905067035.2 | Contig | 26 | 6,249,570 | 3 | 95.1 | 47,495,958 | 11.75 |
| Sar-2-20-1 | *Oryza* | *Oryza sativa* | 2013 | GCA_011799915.1 | Contig | 16 | 6,161,260 | 4 | 98.2 | 46,284,791 | 11.25 |
| AG098 | *Oryza* | *Oryza sativa* | 2011 | GCA_905067015.2 | Contig | 33 | 6,094,221 | 4 | 96.9 | 47,810,826 | 11.83 |
| PR003 | *Oryza* | *Oryza sativa* | 2003 | GCA_905067075.2 | Contig | 16 | 6,063,740 | 4 | 95.7 | 44,615,198 | 10.32 |
| AG032 | *Oryza* | *Oryza sativa* | 2011 | GCA_905067055.2 | Contig | 24 | 5,961,411 | 4 | 96 | 45,916,910 | 11.17 |
| AG059 | *Oryza* | *Oryza sativa* | 2011 | GCA_905066965.2 | Contig | 37 | 5,900,770 | 4 | 97 | 47,743,121 | 12.05 |
| AG038 | *Oryza* | *Oryza sativa* | 2011 | GCA_905067005.2 | Contig | 19 | 5,673,907 | 4 | 95.7 | 46,291,169 | 11.13 |
| FJ98099 | *Oryza* | *Oryza sativa* | 1998 | GCA_011799925.1 | Contig | 10 | 5,637,639 | 4 | 98.3 | 44,637,475 | 10.27 |
| AV1-1-1 | *Oryza* | *Oryza sativa* | 2015 | GCA_011799965.1 | Contig | 13 | 5,503,597 | 4 | 98.2 | 44,970,614 | 10.66 |
| FJ72ZC7-77 | *Oryza* | *Oryza sativa* | 1992 | GCA_011799905.1 | Contig | 13 | 5,454,130 | 3 | 97.8 | 43,369,826 | 10.24 |
| FR13 | *Oryza* | *Oryza sativa* | 1988 | GCA_900474655.3 | Contig | 31 | 5,398,440 | 4 | 98.2 | 46,410,415 | 11.49 |
| AG002 | *Oryza* | *Oryza sativa* | 2010 | GCA_905067045.2 | Contig | 36 | 4,555,161 | 5 | 97 | 46,086,469 | 11.25 |
| FJ81278 | *Oryza* | *Oryza sativa* | 1981 | GCA_002368475.1 | Contig | 54 | 4,134,126 | 5 | 98.2 | 43,846,566 | 10.34 |
| San_Andrea | *Oryza* | *Oryza sativa* | 2001 | GCA_905067085.2 | Contig | 35 | 4,122,582 | 5 | 95.7 | 48,509,714 | 12.1 |
| guy11 | *Oryza* | *Oryza sativa* | 1978 | GCA_002368485.1 | Scaffold | 56 | 3,275,692 | 5 | 98.5 | 42,869,699 | 11.66 |
| Arcadia2 | *Setaria* | *Setaria italica* | 1989 | GCA_012654115.1 | Scaffold | 23 | 5,982,914 | 4 | 78.7 | 45,684,507 | 10.97 |
| US71 | *Setaria* | *Setaria italica* | 1998 | GCA_900474175.3 | Contig | 55 | 2,812,411 | 5 | 98.3 | 45,580,691 | 10.76 |
| LpKY97 | *Lolium* | *Lolium perenne* | 1989 | GCA_012272995.1 | Complete genome | 9 | - | - | 97.9 | 45,612,971 | 6.07 |
| BTJP4-1 | *Triticum* | *Triticum aestivum* | 2016 | GCA_900474225.2 | Contig | 59 | 4,344,896 | 4 | 69.3 | 44,506,711 | 5.55 |
| BTGP6-f | *Triticum* | *Triticum aestivum* | 2017 | GCA_900474435.2 | Contig | 57 | 3,705,381 | 5 | 64.1 | 44,234,332 | 5.22 |
| BTGP1-b | *Triticum* | *Triticum aestivum* | 2017 | GCA_900474635.2 | Contig | 74 | 2,814,025 | 6 | 61.1 | 44,406,101 | 5.25 |
| BTMP13_1 | *Triticum* | *Triticum aestivum* | 2016 | GCA_900474375.2 | Contig | 16 | 6,037,509 | 3 | 56.6 | 43,978,086 | 5.07 |
| B71 | *Triticum* | *Triticum aestivum* | 2012 | GCA_004785725.1 | Chromosome | 13 | 6,442,091 | 3 | 98.6 | 44,516,808 | 6.18 |
| BR32 | *Triticum* | *Triticum aestivum* | 1990 | GCA_900474545.3 | Contig | 17 | 5,096,353 | 3 | 98.3 | 41,805,140 | 4.84 |
| MZ5-1-6 | *Eleusine* | *Eleusine coracana* | 1976 | GCA_004346965.1 | Complete genome | 7 | - | - | 98.5 | 42,703,282 | 6.37 |
| CD156 | *Eleusine* | *Eleusine indica* | 1989 | GCA_900474475.3 | Contig | 27 | 5,531,649 | 4 | 98.1 | 43,939,965 | 5.24 |
| NI907 | *M. grisea* | *Digitaria sanguinalis* | 1974 | GCA_004355905.1 | Chromosome | 43 | 5,912,490 | 3 | 97.1 | 44,557,582 | 4.64 |

**Table S2:** Comparison of TE content for different TE annotation methods. The TE copy number and length in basepairs is shown for *de novo* annotation without filtering for TE-associated domains, compared to our final results with domain filtering, and to using only the RepBase (Bao et al. 2015) fngrep version 25.10 TE library for annotation. Also included is the content of MgSINE (Kachroo et al. 1995), a non-autonomous element previously characterized to have copy number variation in *Magnaporthe* species (Shirke et al. 2016) that is excluded by our domain-filtering method. Entries are colored as a heatmap, with greater values having darker color.

|  |  | TE content without domain filtering | | TE content after domain filtering (final result) | | TE content only using RepBase | | TE content of only MgSINE | |
| --- | --- | --- | --- | --- | --- | --- | --- | --- | --- |
| Genome | Lineage | Copy # | Length (bp) | Copy # | Length (bp) | Copy # | Length (bp) | Copy # | Length (bp) |
| AG006 | *Oryza* | 16243 | 9821745 | 1774 | 5363093 | 2052 | 4652985 | 512 | 163642 |
| AG039 | *Oryza* | 16451 | 9832472 | 1892 | 5582098 | 2203 | 4813281 | 557 | 176276 |
| Sar-2-20-1 | *Oryza* | 16422 | 9263031 | 1804 | 5207862 | 2023 | 4549387 | 489 | 156386 |
| AG098 | *Oryza* | 16868 | 10057138 | 1907 | 5655132 | 2250 | 4960262 | 554 | 174735 |
| PR003 | *Oryza* | 13722 | 8035790 | 1522 | 4602707 | 1817 | 4057322 | 434 | 139664 |
| AG032 | *Oryza* | 15180 | 9026160 | 1708 | 5126914 | 1985 | 4459992 | 486 | 155849 |
| AG059 | *Oryza* | 16823 | 10277338 | 1935 | 5755180 | 2230 | 4984954 | 544 | 169543 |
| AG038 | *Oryza* | 15405 | 9347131 | 1709 | 5153132 | 2021 | 4483132 | 498 | 159876 |
| FJ98099 | *Oryza* | 14593 | 8043998 | 1552 | 4584682 | 1762 | 3984828 | 448 | 143984 |
| AV1-1-1 | *Oryza* | 14992 | 8483890 | 1604 | 4794784 | 1817 | 4091717 | 448 | 145298 |
| FJ72ZC7-77 | *Oryza* | 13510 | 7420081 | 1482 | 4440847 | 1699 | 3891213 | 425 | 137486 |
| FR13 | *Oryza* | 15643 | 9264856 | 1787 | 5332684 | 2080 | 4599822 | 509 | 159769 |
| AG002 | *Oryza* | 14556 | 8886689 | 1672 | 5184887 | 1952 | 4491394 | 483 | 155720 |
| FJ81278 | *Oryza* | 13949 | 7773531 | 1583 | 4534753 | 1817 | 3931304 | 424 | 135962 |
| San_Andrea | *Oryza* | 17258 | 10584644 | 1986 | 5871895 | 2296 | 5109297 | 547 | 171350 |
| guy11 | *Oryza* | 11992 | 7326757 | 1513 | 4997716 | 1971 | 4091473 | 364 | 115703 |
| Arcadia2 | *Setaria* | 13793 | 8522710 | 1290 | 5011497 | 1852 | 3893867 | 250 | 65039 |
| US71 | *Setaria* | 14052 | 8568202 | 1298 | 4904034 | 1761 | 3957366 | 253 | 69610 |
| LpKY97 | *Lolium* | 14533 | 7688540 | 826 | 2769808 | 1106 | 1940796 | 112 | 26732 |
| BTJP4-1 | *Triticum* | 13568 | 7035487 | 747 | 2468944 | 1021 | 1861753 | 120 | 28285 |
| BTGP6-f | *Triticum* | 13549 | 6826557 | 721 | 2310779 | 978 | 1727816 | 123 | 29706 |
| BTGP1-b | *Triticum* | 13661 | 7001010 | 724 | 2329889 | 994 | 1731886 | 117 | 28220 |
| BTMP13_1 | *Triticum* | 13268 | 6715431 | 677 | 2228079 | 946 | 1668586 | 108 | 26479 |
| B71 | *Triticum* | 13430 | 6975700 | 805 | 2749343 | 1105 | 1912244 | 114 | 27332 |
| BR32 | *Triticum* | 10419 | 4827106 | 563 | 2023105 | 838 | 1395959 | 47 | 10380 |
| MZ5-1-6 | *Eleusine* | 11507 | 5762145 | 726 | 2719140 | 1002 | 1897481 | 50 | 10533 |
| CD156 | *Eleusine* | 12682 | 6135266 | 651 | 2303453 | 1047 | 1338243 | 60 | 12062 |
| NI907 | *M. grisea* | 13179 | 5562238 | 718 | 2066070 | 937 | 1579728 | 78 | 17472 |

**Table S3:** Names and classifications of TEs discussed. The name we used throughout the paper for each TE family is shown, along with its original name in RepBase (Bao et al. 2015) fngrep version 25.10, the class, and the superfamily each element belongs to. We adopted a naming convention for *Ty3* (formerly *Gypsy*) elements, where any “*GY*” in the RepBase name was replaced with “*Ty3*” in order to use a non-discriminatory and respectful naming scheme (Wei et al. 2022). *Grasshopper* is the original name of the *GYPSY1* RepBase element, so it is used instead (Dobinson 1993). Elements that did not correspond to a specific family in RepBase are indicated by “N/A,” and are named by their superfamily (i.e. *Copia_elem*). LTR = long terminal repeat retrotransposon, NLTR = non-LTR retrotransposon, DNA = DNA transposon.

| **TE family name used** | **RepBase name** | **Class** | **Superfamily** |
| --- | --- | --- | --- |
| *Ty3_MAG1* | *GYMAG1* | LTR | *Ty3* |
| *Ty3_MAG2* | *GYMAG2* | LTR | *Ty3* |
| *Grasshopper (Grh)* | *GYPSY1* | LTR | *Ty3* |
| *MAG_Ty3* | *MAGGY* | LTR | *Ty3* |
| *MGRL3* | *MGRL3* | LTR | *Ty3* |
| *PYRET* | *PYRET* | LTR | *Ty3* |
| *Copia_elem* | N/A | LTR | *Ty1/Copia* |
| *MGR583* | *MGR583* | NLTR | *LINE/Tad1* |
| *MoTeR1* | *MoTeR1* | NLTR | *LINE/CRE* |
| *POT2* | *POT2* | DNA | *Tc1/Mariner* |
| *TcMar_elem* | N/A | DNA | *Tc1/Mariner* |


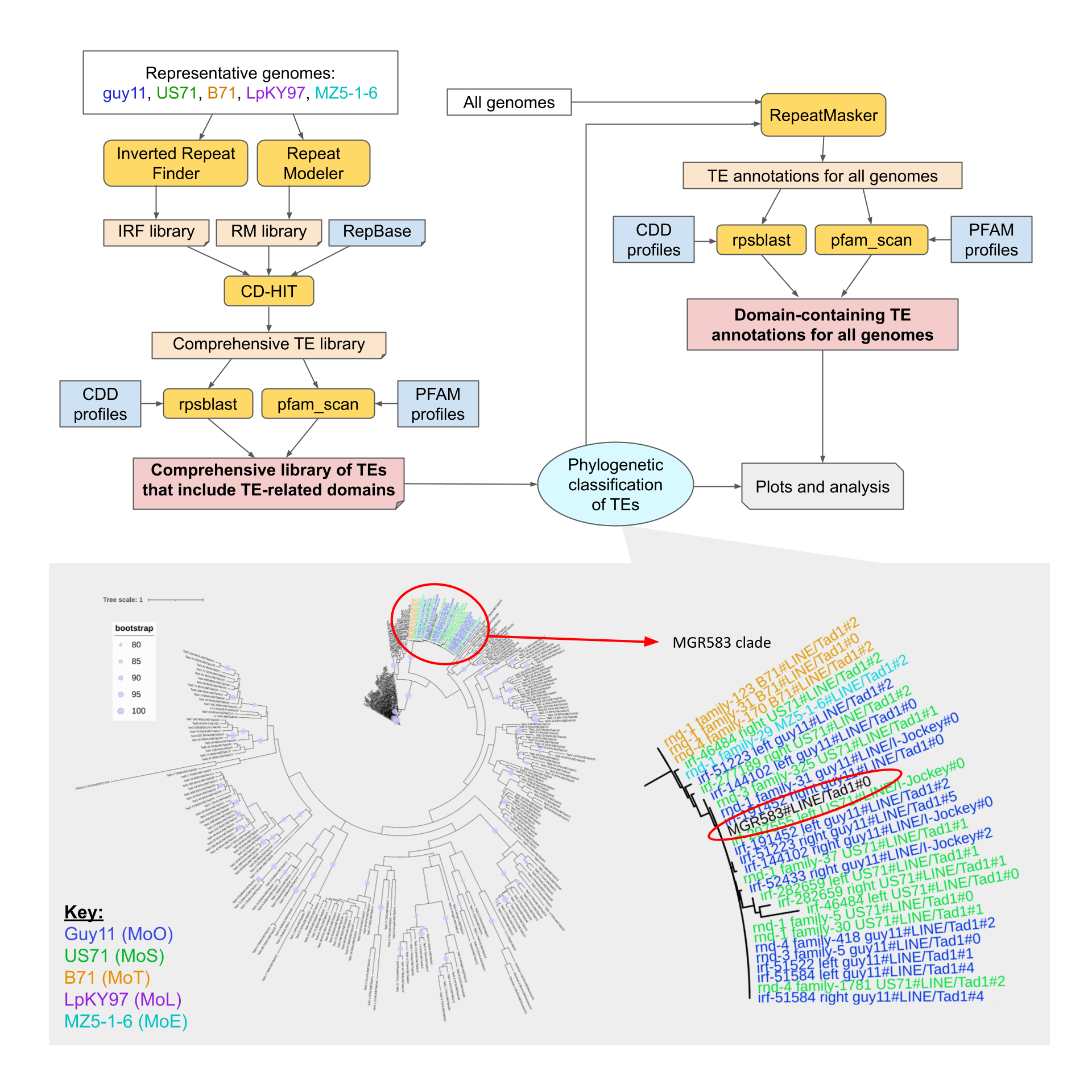


**Figure S1:** TE annotation pipeline diagram. The five representative genomes, Guy11 (MoO), US71 (MoS), B71 (MoT), LpKY97 (MoL), and MZ5-1-6 (MoE) were used for *de novo* TE annotation to produce an unbiased TE library that is representative of TE content in all lineages. The bottom gray box provides an example of how TEs were classified. Shown is a tree based on the Exo_endo_phos_2 domain with a phylogenetically defined TE subclade indicated by the red circle. Subclades of *de novo* elements (in color) that grouped with a known RepBase element (*MGR583* in this example, circled in red) were classified as that element’s family. Colored text names of *de novo* elements represent the genome they were annotated in, as shown in the key.


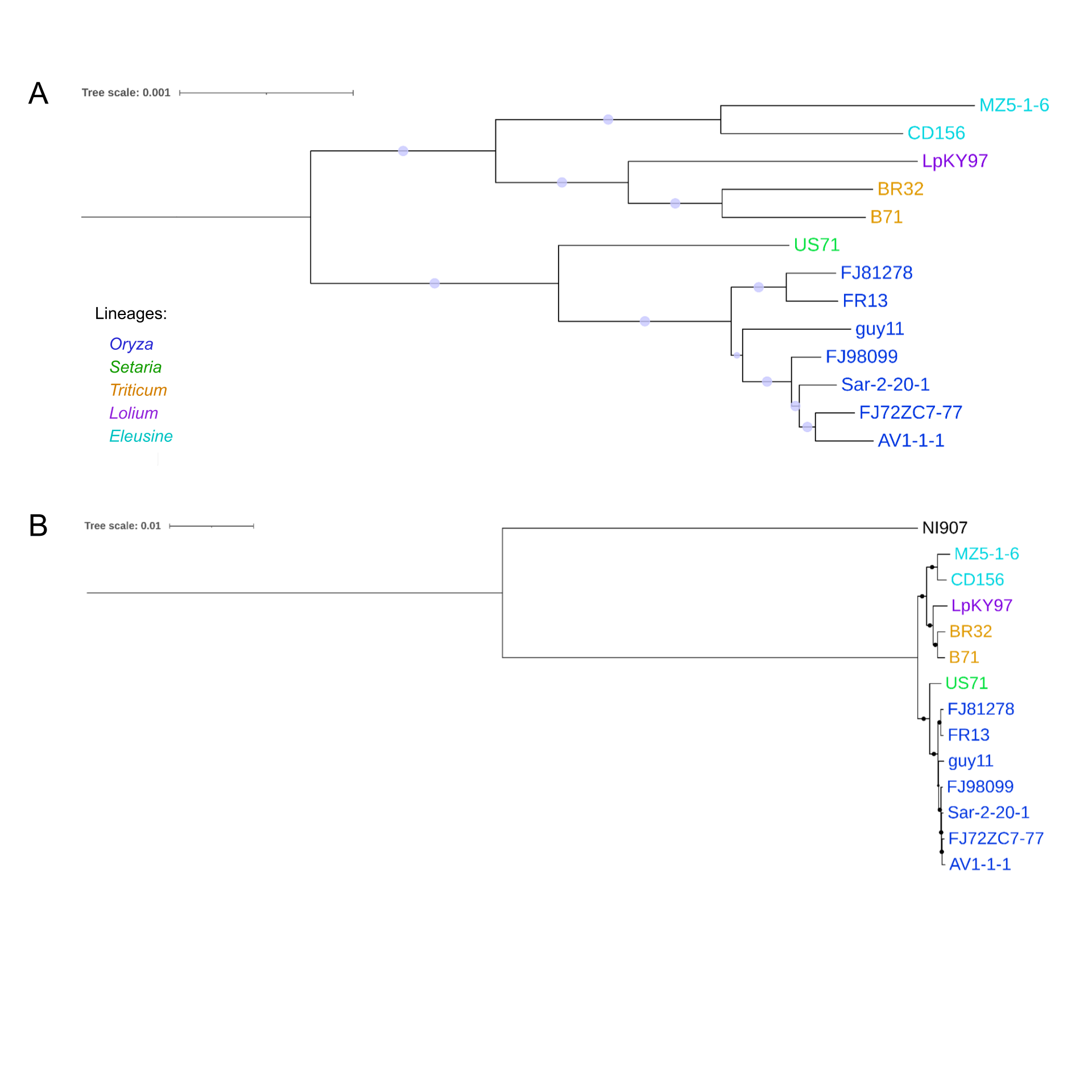


**Figure S2:** Maximum-likelihood (ML) phylogeny of *M. oryzae* genomes based on the alignment of 8,655 single copy orthologous genes (SCOs), **A,** zoomed in without outgroup and **B,** including *Magnaporthe grisea* outgroup (NI907). Only genomes with BUSCO score greater than 97% were included. Bootstrap value of 1 is indicated by circles on the branches.


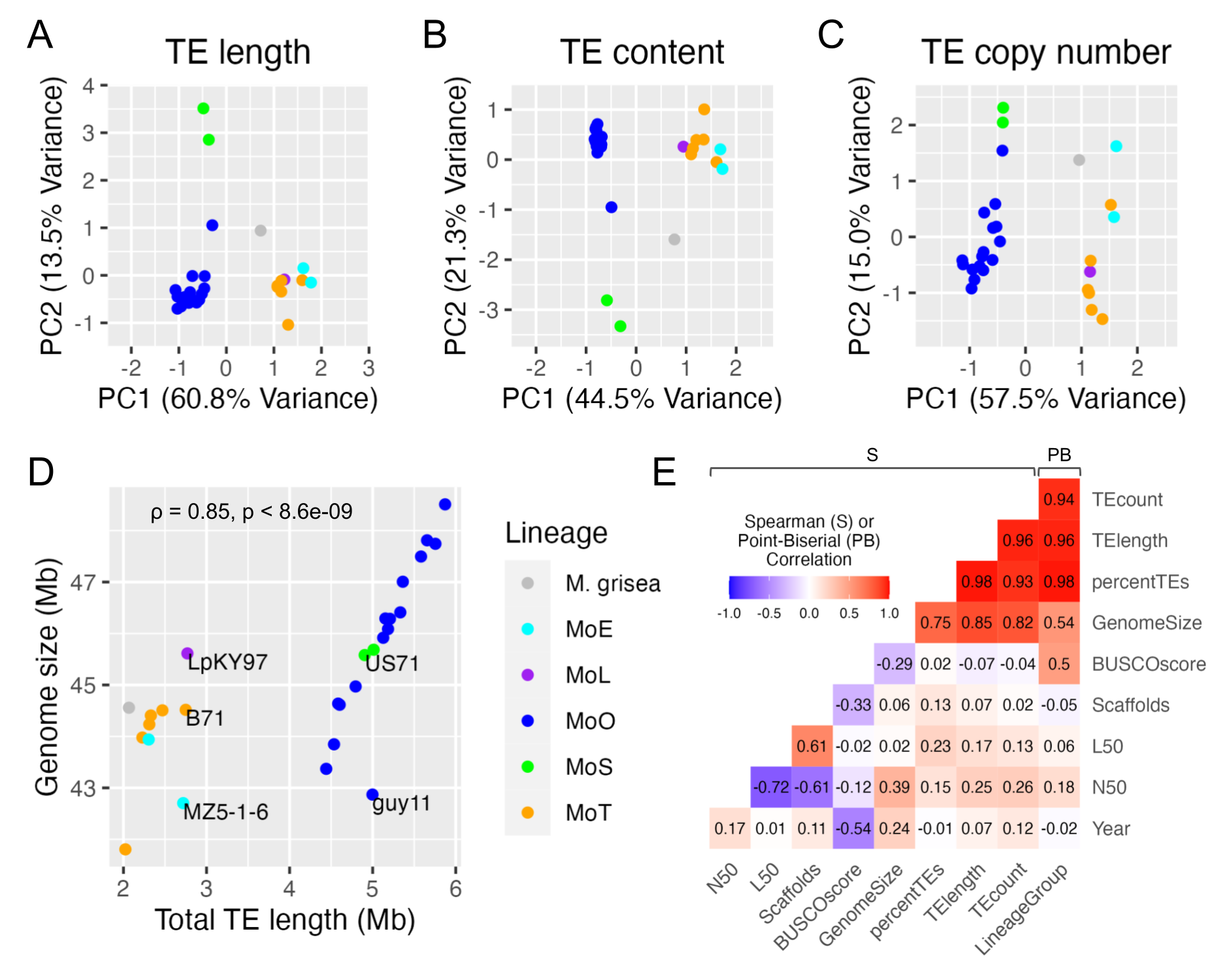


**Figure S3:** TE content clearly differentiates MoO-MoS versus MoT-MoL-MoE lineage groups, while other variables have weaker or no correlation to lineage identity. Scatterplots of principal components (PCs) 1 and 2 values from principal component analyses (PCAs) are shown, for **A,** the length that each TE occupies in each genome, **B,** the percentage of each TE’s copy number out of the total TE count in each genome, and **C,** the copy number of each TE in each genome. Each point represents one genome, and the percentage of variation that each PC describes is shown on the axes. **D,** The correlation between total TE length (Mb) and genome size (Mb) is shown, with Spearman’s ρ (rho) and p-value. **E,** Correlation matrix, where Point-Biserial correlation coefficients were calculated between binary (LineageGroup) and continuous (all other) variables, and Spearman correlation coefficients were calculated between all pairs of continuous variables.


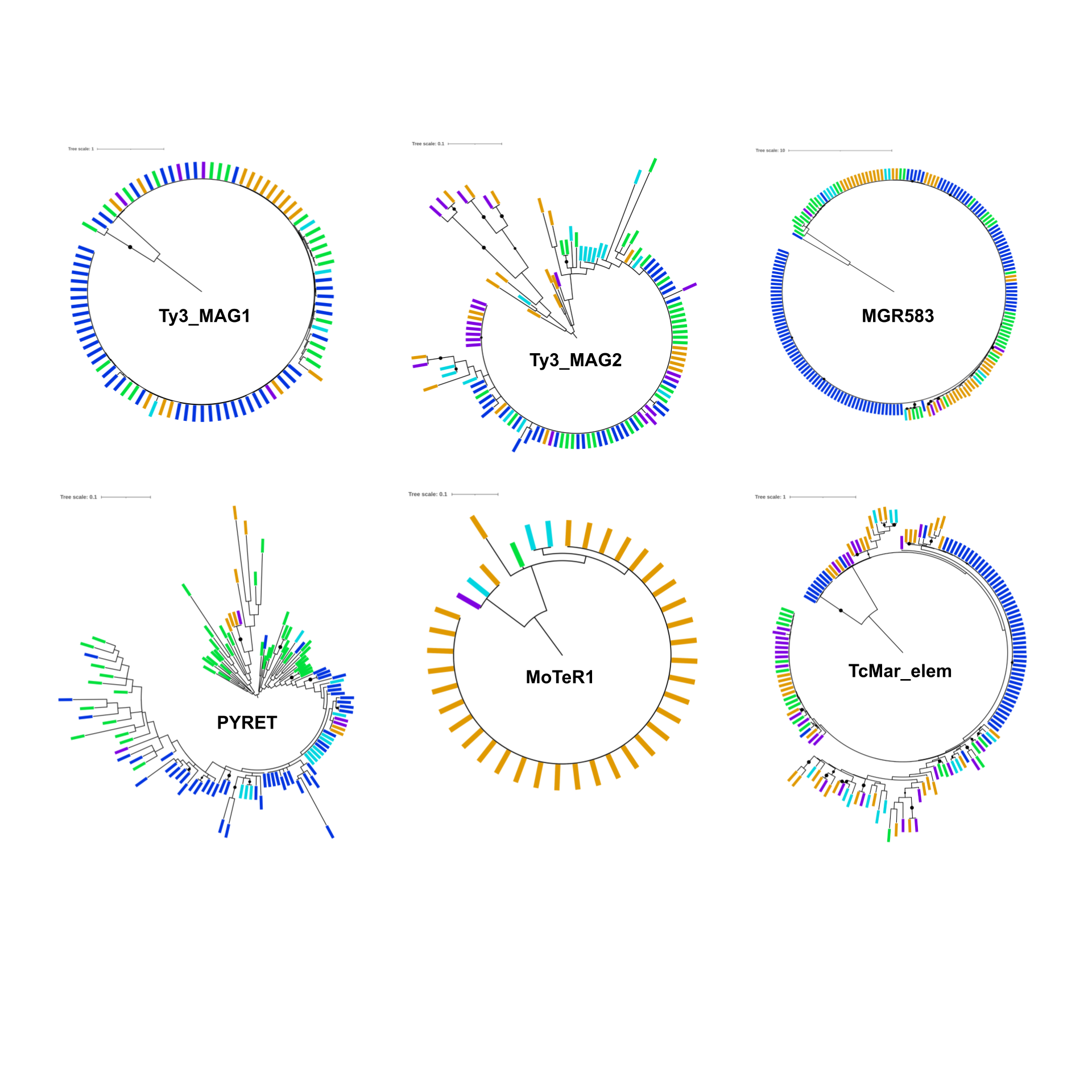


**Figure S4:** Domain-based maximum-likelihood (ML) phylogenies for *Ty3_MAG1*, *Ty3_MAG2*, *MGR583*, *PYRET*, *MoTeR1*, and *TcMar_elem*. Colored rectangle tips correspond to the genome each element is from: blue=Guy11 (MoO), green=US71 (MoS), orange=B71 (MoT), purple=LpKY97 (MoL), and cyan=MZ5-1-6 (MoE). Black circles indicate bootstrap value ≥80.


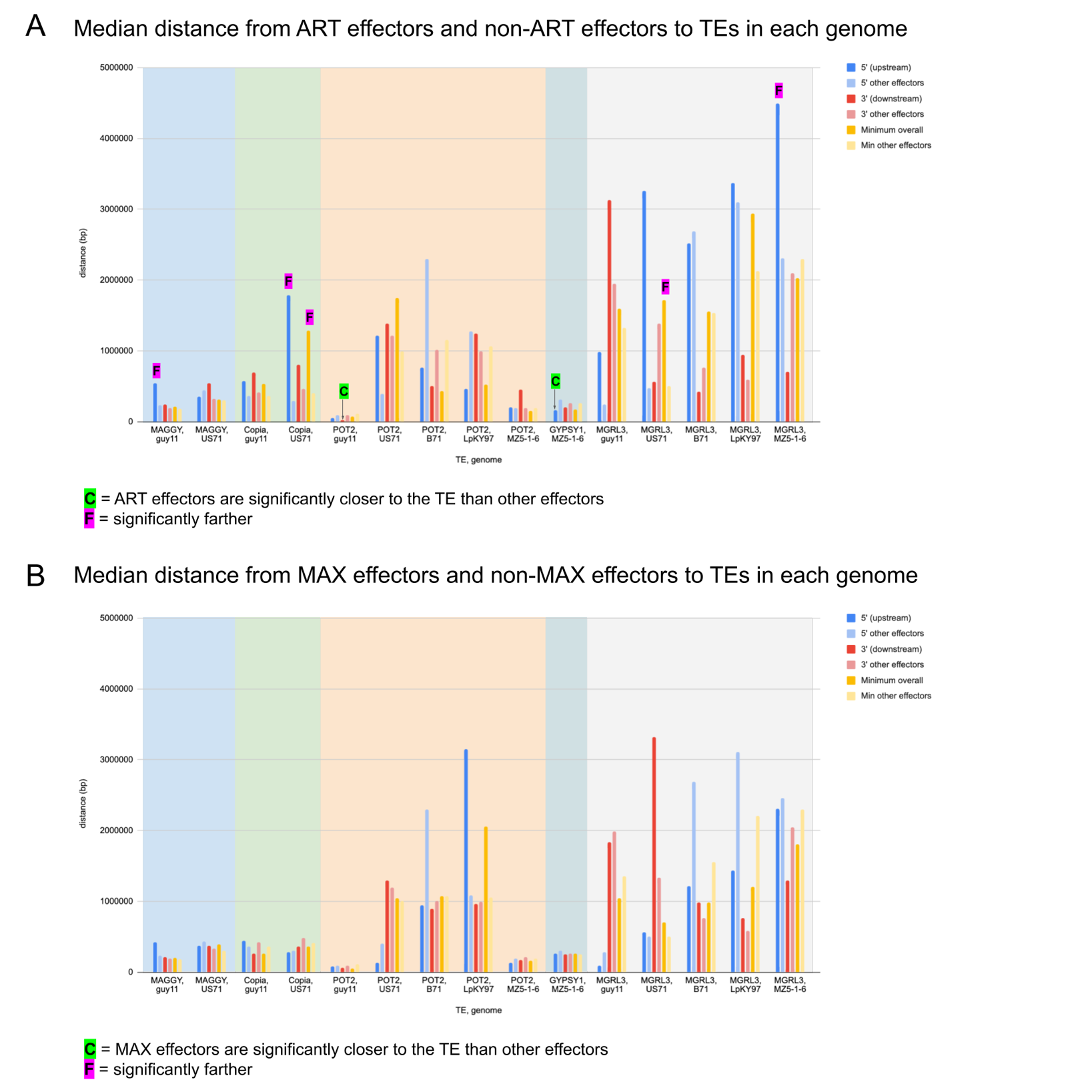


**Figure S5:** Expanded ART & MAX effectors distance to TEs, compared to other effectors. The median distance from **A,** ART and **B,** MAX effectors to various TEs is shown. Permutation tests of the difference in median distance, with 1,000 replicates, were performed for the 5’ distance, 3’ distance, and minimum distance on either side of each effector. Significant values from the permutation test are indicated, where C=significantly closer to TEs, and F=significantly farther from TEs.


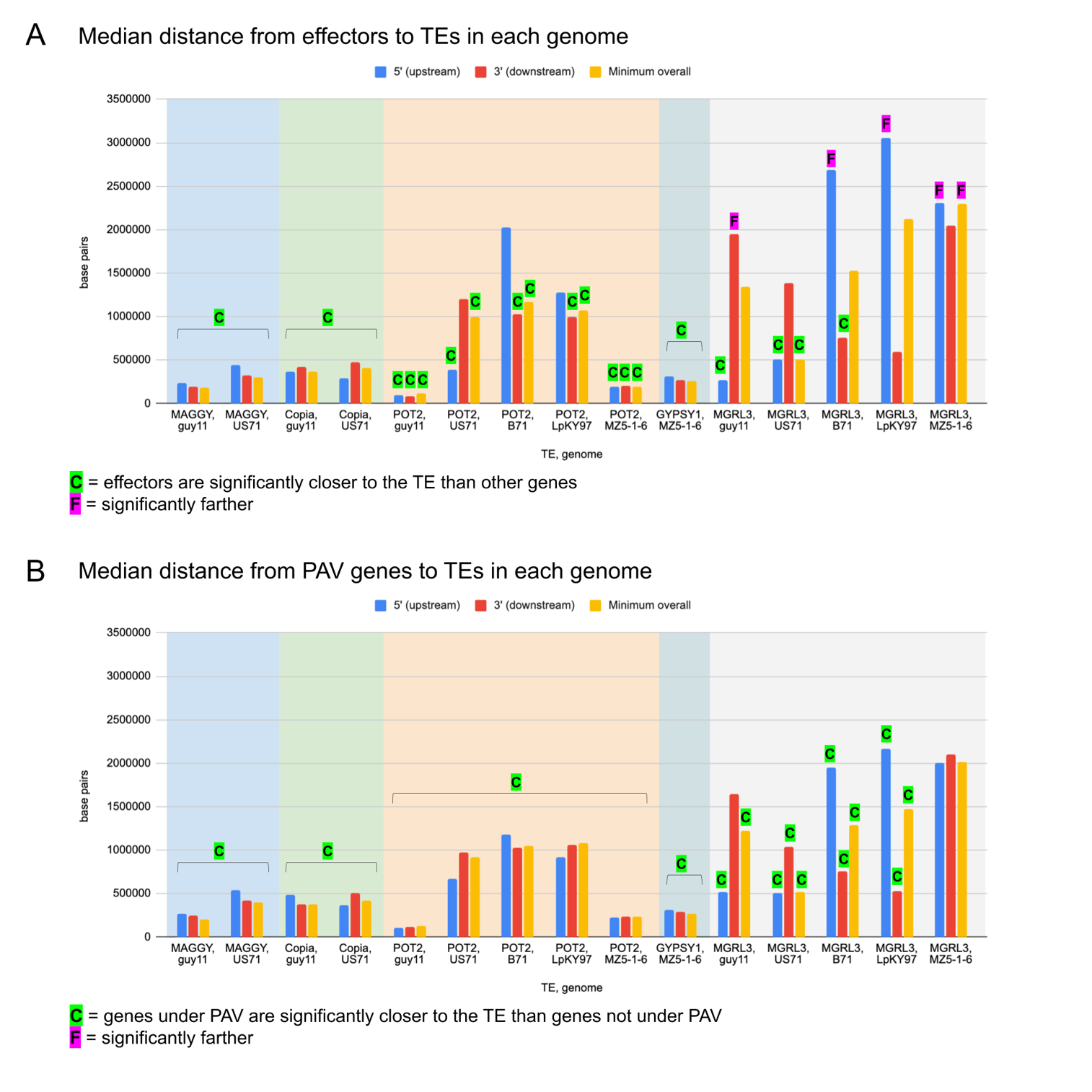


**Figure S6:** Effectors and genes under presence-absence variation (PAV) distance to TEs, compared to all other genes. The median distance from **A,** effectors and **B,** genes under PAV to various TEs is shown. Permutation tests of the difference in median distance, with 1,000 replicates, were performed for the 5’ distance, 3’ distance, and minimum distance on either side of each effector. Significant values from the permutation test are indicated, where C=significantly closer to TEs, and F=significantly farther from TEs.


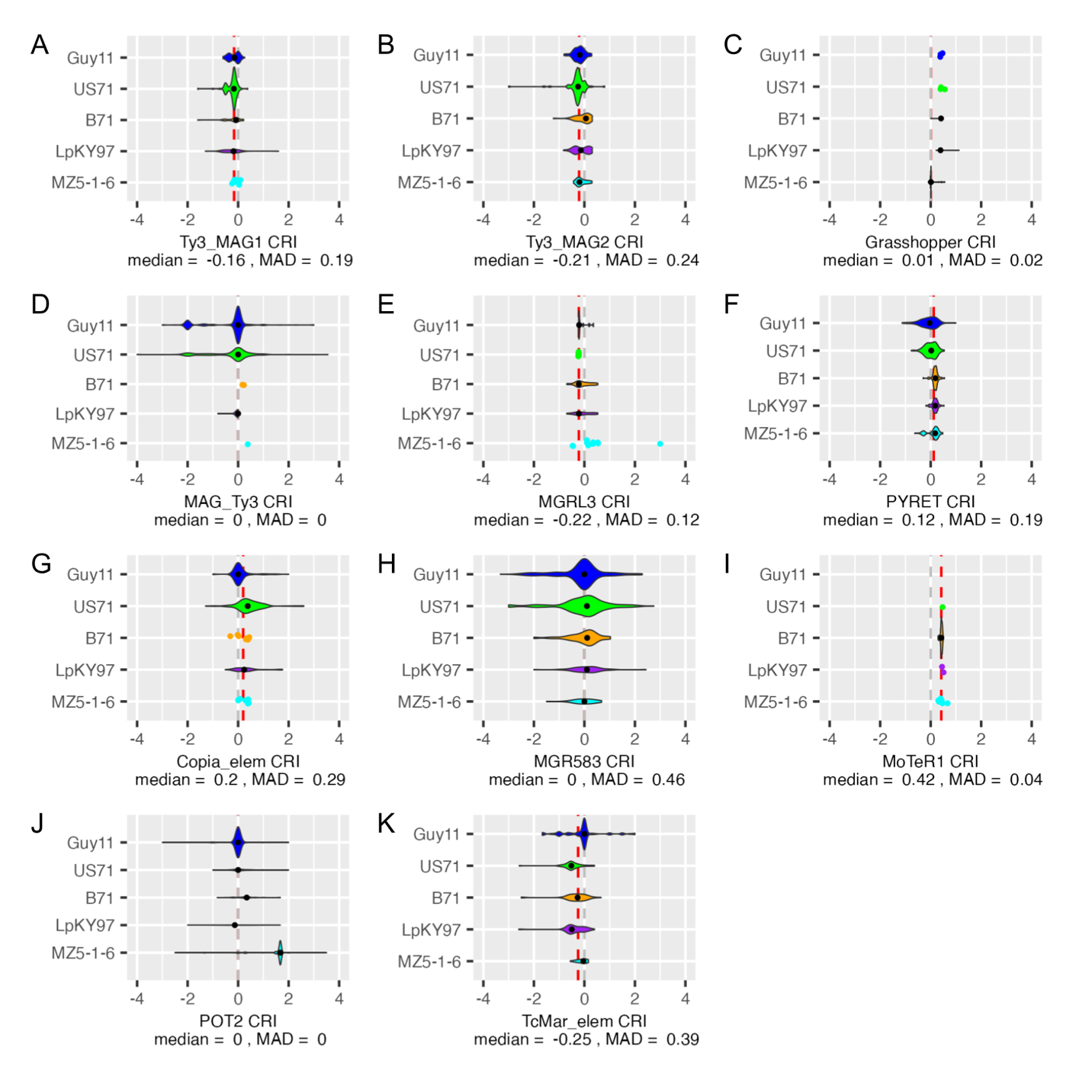


**Figure S7:** Composite RIP Index (CRI) per element in each genome, where CRI > 0 indicates RIP and CRI ≤ 0 indicates no RIP. The CRI of each TE family in each representative genome is shown for **A,** *Ty3_MAG1*, **B,** *Ty3_MAG2*, **C,** *Grasshopper*, **D,** *MAG_Ty3*, **E,** *MGRL3*, **F,** *PYRET*, **G,** *Copia_elem*, **H,** *MGR583*, **I,** *MoTeR1*, **J,** *POT2*, and **K,** *TcMar_elem*. Data is shown as a violin plot unless there are <10 points, in which case a jitterplot was used. Within each plot, violin width is proportional to the number of TE copies represented. Dashed lines indicate: grey = 0 CRI cutoff, red = the median CRI of the TE. The median CRI of the TE and the median absolute deviation (MAD) are specified.


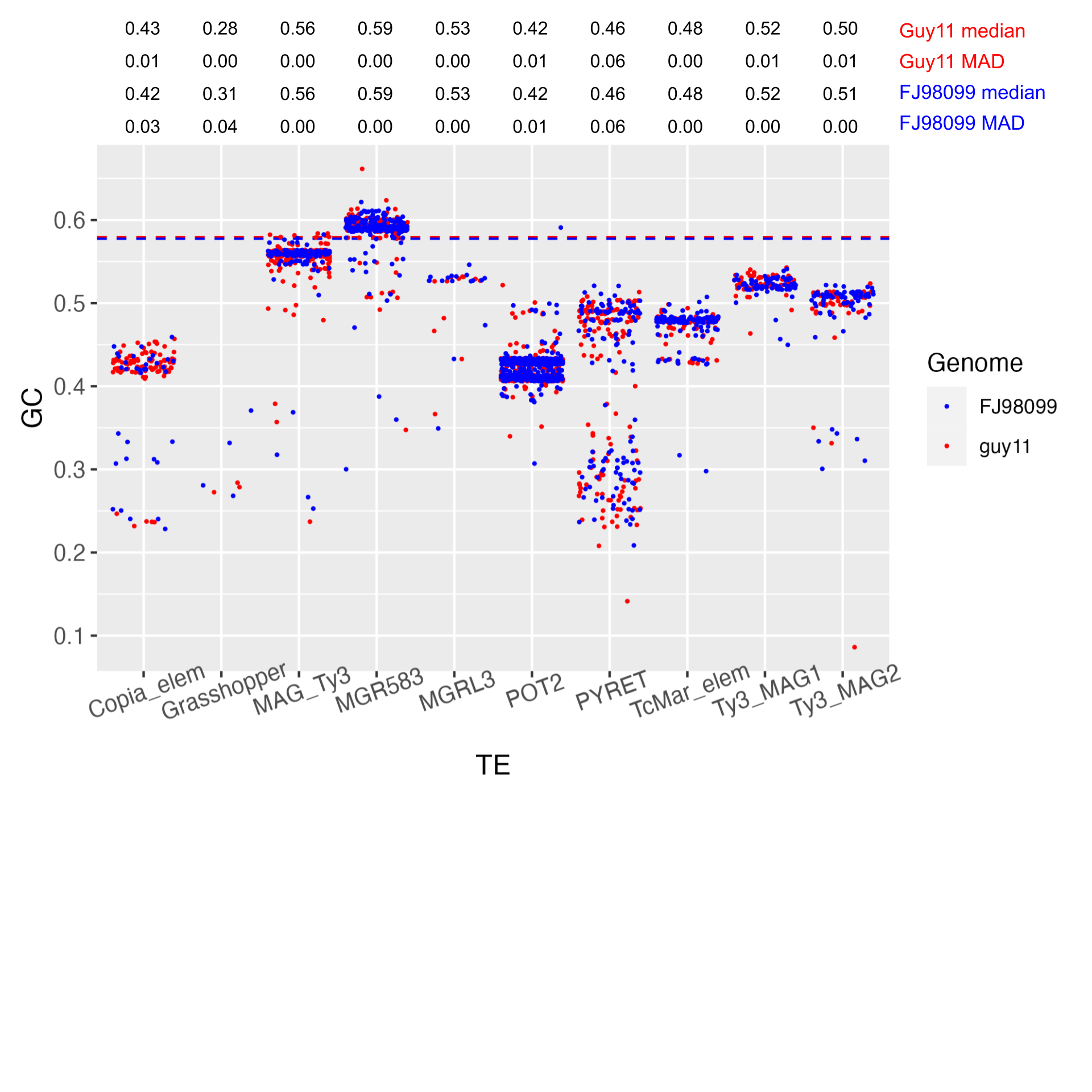


**Figure S8:** Jitter-plot showing GC content in each TE family, for the recombining Guy11 genome (red) and the clonal FJ98099 genome (blue). Each dot represents one TE copy, and dashed lines represent the genome-wide average GC-content of coding regions for each genome. Median GC content and median absolute deviation (MAD) values are displayed above for each TE in both genomes.


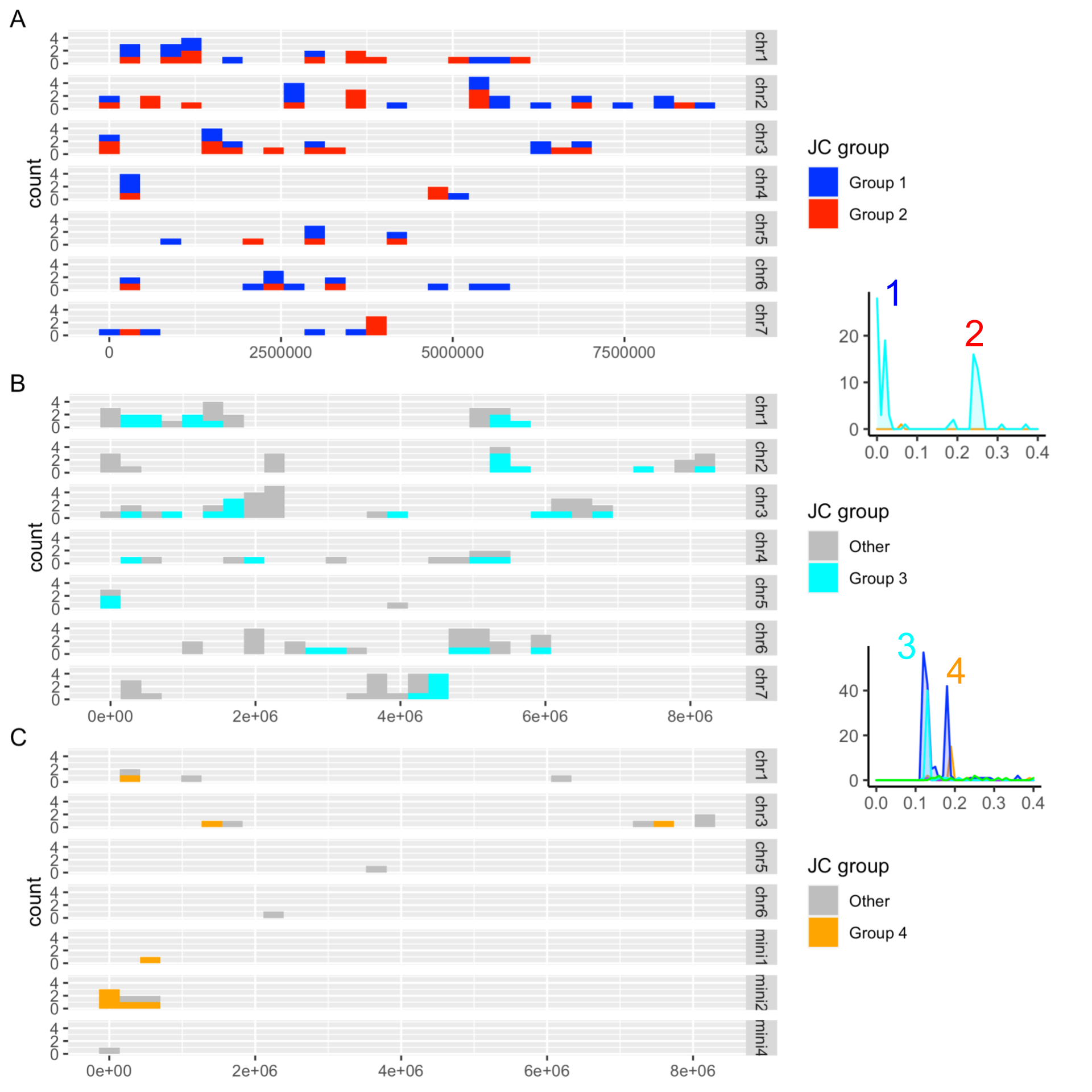


**Figure S9:** Localization of expanded TEs in *M. oryzae* genomes. **A,** The location of *Grasshopper* elements of lower (Group 1 in red) and higher (Group 2 in blue) Jukes-Cantor distance throughout MZ5-1-6’s seven chromosomes. The Jukes-Cantor plot for *Grasshopper* with groups 1 and 2 peaks labeled is shown for reference. **B,** The location of *POT2* elements in MZ5-1-6 that group with the lower Guy11 *POT2* Jukes-Cantor peak (Group 3 in cyan). **C,** The location of *POT2* elements in B71 that group with the higher Guy11 *POT2* Jukes-Cantor peak (Group 4 in orange). The Jukes-Cantor plot for *POT2* with groups 3 and 4 peaks labeled is shown for reference.


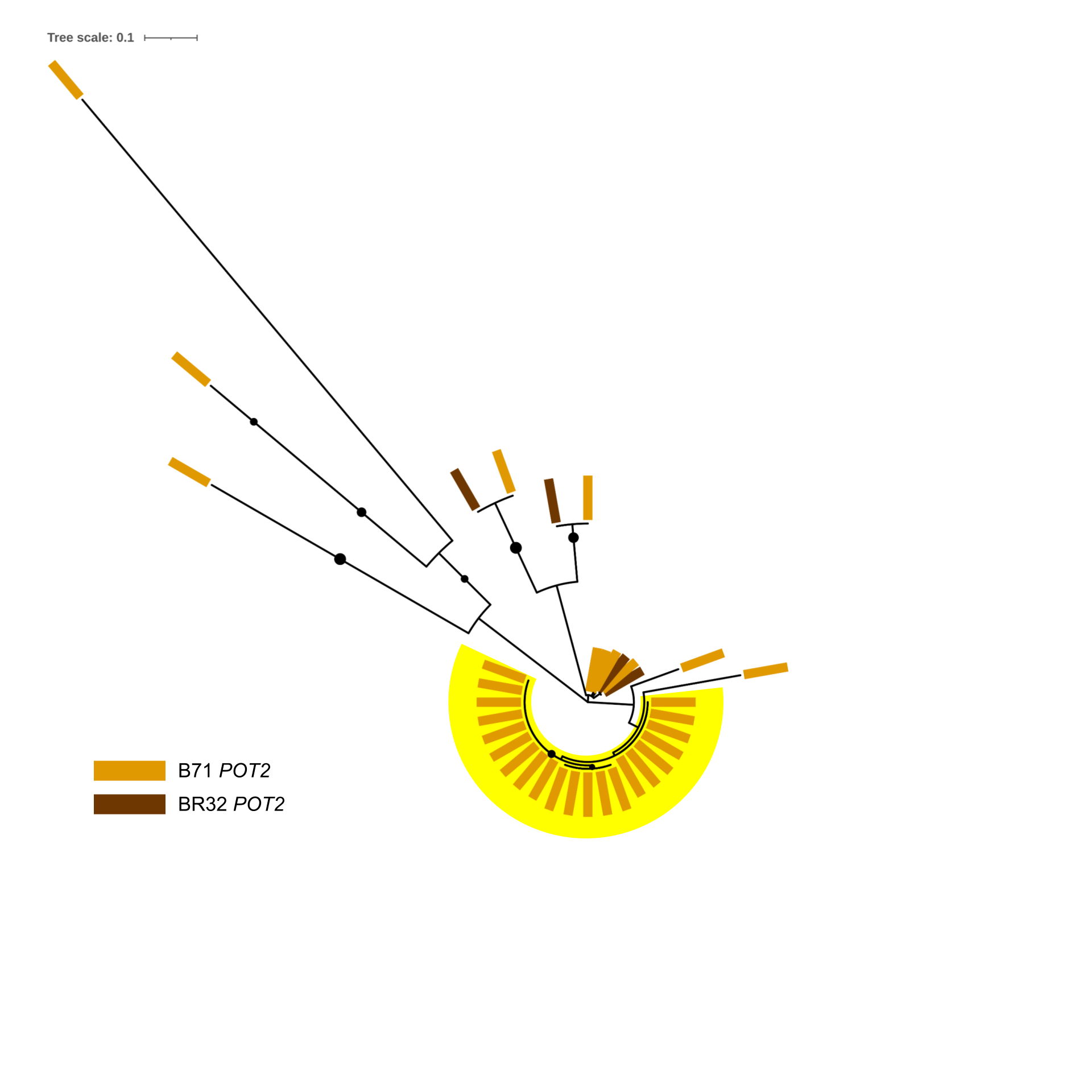


**Figure S10:** Domain-based maximum-likelihood (ML) phylogeny of *POT2* from MoT genomes B71 and BR32. The yellow highlighted clade corresponds to the potentially transferred B71 *POT2* clade from Figure 5A. Black circles indicate bootstrap values of ≥80.


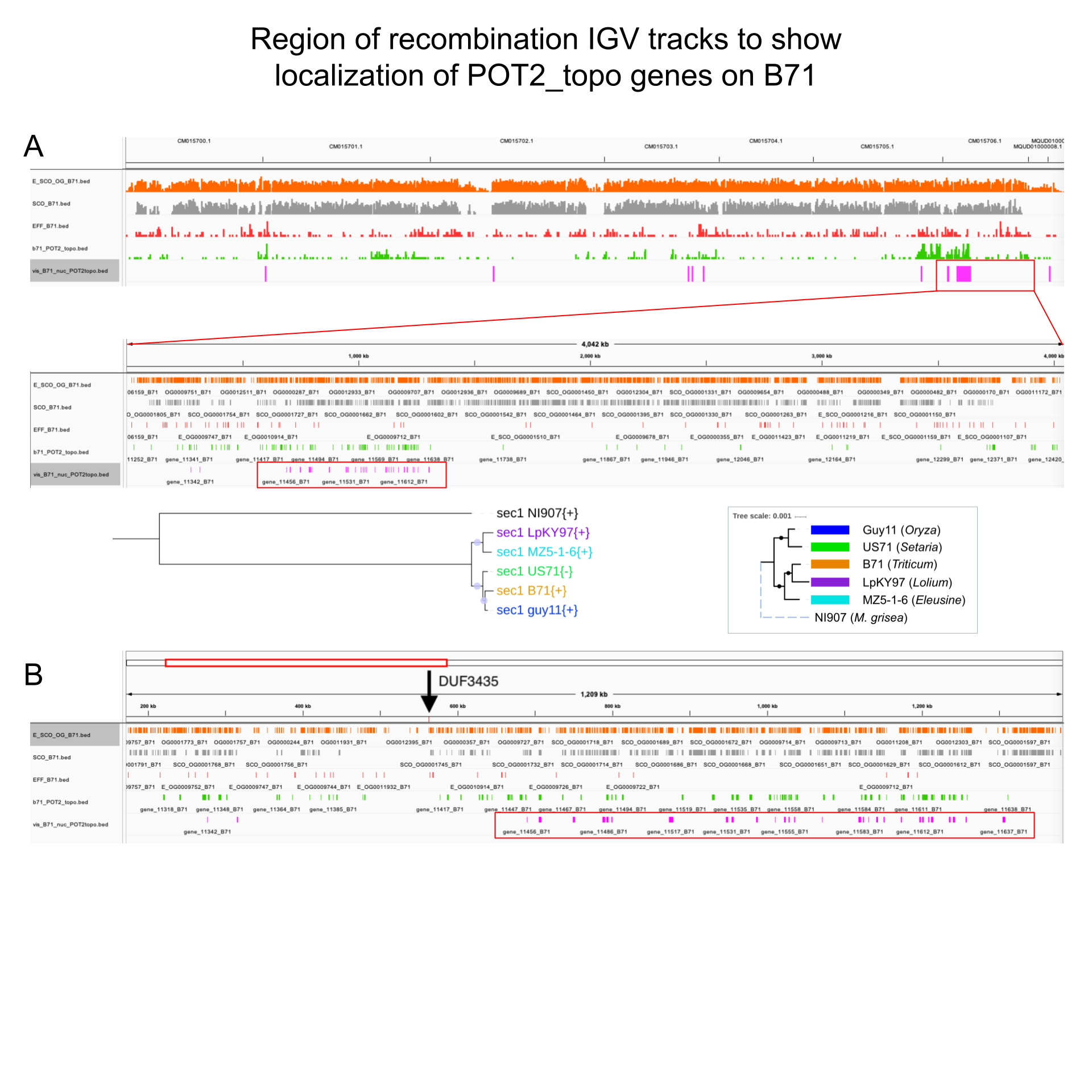


**Figure S11:** Genes following *POT2* tree topology are localized in a region on B71’s chromosome 7. **A,** The first IGV track shows all seven chromosomes, and the second track shows just chromosome 7. The tree on the left side shows the phylogeny constructed from an alignment of the full-length region in each isolate, and the genome tree is shown on the right for comparison. **B,** A gene containing a fragmented DUF3435 domain, which is associated with *Starship* elements, is located nearby and upstream of the region, indicated by the black arrow. The locations of genes that follow the *POT2* tree topology are indicated in pink, and the entire region is boxed in red in each track.

**References**

Bao W, Kojima KK, Kohany O. 2015. Repbase Update, a database of repetitive elements in eukaryotic genomes. *Mob. DNA* 6:11.

Dobinson KF. 1993. *Grasshopper,* a Long Terminal Repeat (LTR) Retroelement in the Phytopathogenic Fungus *Magnaporthe grisea*. *Mol. Plant. Microbe Interact.* 6:114.

Kachroo P, Leong SA, Chattoo BB. 1995. Mg-SINE: a short interspersed nuclear element from the rice blast fungus, Magnaporthe grisea. Proc. Natl. Acad. Sci. 92:11125–11129.

Shirke MD, Mahesh HB, Gowda M. 2016. Genome-Wide Comparison of Magnaporthe Species Reveals a Host-Specific Pattern of Secretory Proteins and Transposable Elements. PLOS ONE 11:e0162458.

Wei K, Aldaimalani R, Mai D, Zinshteyn D, Prv S, Blumenstiel JP, Kelleher ES, Brooks E. 2022. Rethinking the “gypsy” retrotransposon: A roadmap for community-driven reconsideration of problematic gene names. Open Science Framework Available from: https://osf.io/fma57
