# Supplementary material for: Intraspecific Variation of Transposable Elements Reveals Differences in the Evolutionary History of Fungal Phytopathogen Pathotypes
Source: Genome Biol Evol. 2023 Nov 17;15(12):evad206. doi: 10.1093/gbe/evad206 (PMC10691877; doi:10.1093/gbe/evad206)
Supplement: evad206_Supplementary_Data [file evad206_supplementary_data.zip › Supplemental Material Legends.docx]

**Supplemental Material**

Additional File 1: Supplementary Figures and Tables.

Table S1: *M. oryzae* genomes used in this study and their metadata.

Table S2: Comparison of TE content for different TE annotation methods.

Table S3: Names and classifications of TEs discussed.

Figure S1: TE annotation pipeline diagram.

Figure S2: Maximum-likelihood (ML) phylogeny of *M. oryzae* genomes based on the alignment of 8,655 single copy orthologous genes (SCOs).

Figure S3: TE content clearly differentiates MoO-MoS versus MoT-MoL-MoE lineage groups, while other variables have weaker or no correlation to lineage identity.

Figure S4: Domain-based maximum-likelihood (ML) phylogenies for *Ty3_MAG1*, *Ty3_MAG2*, *MGR583*, *PYRET*, *MoTeR1*, and *TcMar_elem*.

Figure S5: Expanded ART & MAX effectors distance to TEs, compared to other effectors.

Figure S6: Effectors and genes under presence-absence variation (PAV) distance to TEs, compared to all other genes.

Figure S7: Composite RIP Index (CRI) per element in each genome.

Figure S8: Jitter-plot showing GC content in each TE family, for the recombining Guy11 genome and the clonal FJ98099 genome.

Figure S9: Localization of expanded TEs in *M. oryzae* genomes.

Figure S10: Domain-based maximum-likelihood (ML) phylogeny of *POT2* from MoT genomes B71 and BR32.

Figure S11: Genes following *POT2* tree topology are localized in a region on B71’s chromosome 7.

Additional File 2: Copy number of all TE families in each representative genome.

Additional File 3: GO terms output from PANNZER, filtered for >0.6 PPV value, for genes in the region on chromosome 7 following *POT2* topology.

Additional File 4: PFAM terms output from pfam_scan, filtered for E-value <0.01, for genes in the region on chromosome 7 following *POT2* topology.
